# TGF-β in tumor development and progression: mechanisms and therapeutics

**DOI:** 10.1186/s43556-026-00403-w

**Published:** 2026-01-30

**Authors:** Jialing Liu, Yiwei Wang, Chao Tang, Lulu Zhang, Sidong Xiong, Jun Wang, Chunsheng Dong

**Affiliations:** 1 The Fourth Affiliated Hospital of Soochow University, Institutes of Biology and Medical Science, Jiangsu Key Laboratory of Infection and Immunity, MOE Key Laboratory of Geriatric Diseases and Immunology, Suzhou Medical College of Soochow University, Soochow University, Suzhou, China; 2 Institutes of Biology and Medical Sciences, Jiangsu Key Laboratory of Infection and Immunity, MOE Key Laboratory of Geriatric Diseases and Immunology, Suzhou Medical College of Soochow University, Soochow University, Suzhou, China

**Keywords:** TGF-β signaling, Tumor microenvironment, Metastasis, Anti-tumor immunity, TGF-β-targeting therapy

## Abstract

Transforming growth factor beta (TGF-β) is a pleiotropic cytokine and participates in multiple cellular processes, such as cell development, proliferation, epithelial mesenchymal transition (EMT), and immune responses through SMAD-dependent or SMAD-independent signaling pathways. Notably, TGF-β signaling plays a dual role in tumors, acting as a potent tumor suppressor during early tumorigenesis by inducing apoptosis or cell-cycle arrest while promoting tumor transformation, progression and metastasis in advanced stage through multidimensional mechanisms. Moreover, it is abundant and functions as a master immune checkpoint in the tumor microenvironment (TME), fostering the development of numerous targeted therapies to rectify its aberrant activity in tumors in the past decades. Thus, a comprehensive overview of the pathologic roles, molecular mechanisms and therapeutic potentials of TGF-β signaling in tumors will benefit both the basic and clinical cancer research. Here, we review the complex biology and context-dependent functions of the TGF-β superfamily in regard to tumor, highlighting how it regulates the latter’s development, growth, and dissemination by mainly targeting tumor cells, tumor-associated fibroblasts and various immune cells. We also summarize recent advances in the preclinical and clinical development of different types of TGF‑β‑targeting agents, and discuss their therapeutic potentials and challenges as well as approaches to improve the safety and efficacy of TGF-β pathway-targeted therapy in cancers. Through the summary of known knowledge and the latest updates, this review may provide a general picture on the biological functions of TGF-β in tumors, and facilitate the clinical implications of TGF-β-targeted therapy in tumor patients.

## Introduction

Biological signaling orchestrates fundamental physiological processes, including cell activation, growth, differentiation, and metabolism, commonly through the interaction of soluble/surface molecules and membrane-bound receptors. Among these mediators, TGF-β, a pleiotropic cytokine, exerts diverse functions in different cell types upon binding to its cell surface receptors, and thereby plays a critical role in both health and disease. As such, the roles of the TGF-β signaling and therapeutic potential of TGF-β-targeting therapies in physiological and various pathological conditions have become a major focus in both basic and clinical research fields.

In regard to tumor development and progression, TGF-β exerts a context-dependent anti- or pro-tumor effect. In normal cells and tissues, activated TGF-β signaling mainly functions as a G1-S cell-cycle checkpoint to regulate the survival, proliferation, and genome integrity of cells, thereby effectively restricting the malignant transformation [1, 2]. However, many factors, such as gene mutations and abnormal signaling pathways in the microenvironment, counteract the anti-proliferative effect of TGF-β while retaining its transforming function, eventually resulting in tumorigenesis. In addition, it has been well established that overactivated TGF-β signaling in the tumor microenvironment (TME) is associated with enhanced tumor growth and metastasis, impaired anti-tumor responses, and poor prognosis in cancer patients [3]. Of note, many preclinical and clinical studies have demonstrated substantial anti-tumor potential of TGF-β-targeting agents. However, owing to the high pleiotropy of TGF-β, systemic inhibition of this cytokine tends to exert off-target effects in healthy tissues, triggering adverse events and safety concerns that pose significant obstacles to its clinical translation. Thus, elucidating the molecular mechanisms of TGF-β signaling in tumorigenesis and progression holds promise for improving the safety and efficacy of existing cancer therapies.

In this review, we mainly focus on the regulation of TGF-β signaling and its impact on tumor development, growth, metastasis, as well as anti-tumor immunity. Moreover, we also summarize the types, challenges, and applications of therapeutics targeting the TGF-β pathway in cancer treatment either as monotherapy or combination therapy.

## Overview of TGF-β superfamily: ligands, receptors, and core signaling pathways

The TGF-β superfamily contains more than 30 members, which, based on the structural and biological properties, have been categorized into the TGF-β/NODAL subfamily and the bone morphogenetic protein (BMP) subfamily. In mammals, the TGF-β/NODAL subfamily is composed of TGF-β1/2/3 (collectively known as TGF-β), NODAL, four ACTIVINs (ACTIVIN A/B/C/E), and five growth and differentiation factors (GDFs, GDF1/3/8/9/11), as well as two blocking ligands (INHIBIN and LEFTY) that inhibit the ACTIVIN/NODAL receptors. The BMP subfamily comprises eleven BMPs (BMP2-10&15), four GDFs (GDF5-7&10) and the anti-muellerian hormone (AMH) that associates with gonadal development [4] (Table 1). TGF-β family proteins play crucial roles in multiple cellular processes, such as differentiation, apoptosis, epithelial mesenchymal transition (EMT), angiogenesis, and immune regulation [5, 6].

**Table 1:** TGF-β family proteins and receptors in mammals

TGF-β Ligands		Type I Receptor	Type II Receptor	SMAD
NODAL		ACVR1B, ACVR1C	ACVR2A, ACVR2B	SMAD2/3
TGF-β	TGF-β1	TGFBR1	TGFBR2	SMAD2/3
	TGF-β2	TGFBR1	TGFBR2	SMAD2/3
	TGF-β3	TGFBR1	TGFBR2	SMAD2/3
ACTIVINS	ACTIVIN A	ACVR1B, ACVR1C	ACVR2A, ACVR2B	SMAD2/3
	ACTIVIN B	ACVR1B, ACVR1C	ACVR2A, ACVR2B	SMAD2/3
	ACTIVIN C	ACVR1B, ACVR1C	ACVR2A, ACVR2B	SMAD2/3
	ACTIVIN E	ACVR1B, ACVR1C	ACVR2B	SMAD2/3
BMPs	BMP2	BMPR1A, BMPR1B	ACVR2A, ACVR2B, BMPR2	SMAD1/5
	BMP3	BMPR1A, BMPR1B	ACVR2A, ACVR2B, BMPR2	SMAD1/5
	BMP4	BMPR1A, BMPR1B	ACVR2A, ACVR2B, BMPR2	SMAD1/5
	BMP5	ACVR1A, BMPR1A, BMPR1B	ACVR2A, ACVR2B, BMPR2	SMAD1/5
	BMP6	ACVR1A, BMPR1A, BMPR1B	ACVR2A, ACVR2B, BMPR2	SMAD1/5
	BMP7	ACVR1A, BMPR1A, BMPR1B	ACVR2A, ACVR2B, BMPR2	SMAD1/5
	BMP8	ACVR1A, BMPR1A, BMPR1B	ACVR2A, ACVR2B, BMPR2	SMAD1/5
	BMP8B	BMPR1A, BMPR1B	ACVR2A, BMPR2	SMAD1/5
	BMP9/GDF2	ACVRL1	ACVR2, BMPR2	SMAD1/5
	BMP10	ACVRL1	ACVR2, BMPR2	SMAD1/5
	BMP15	BMPR1B	BMPR2	SMAD1/5
GDFs	GDF1	ACVR1B, ACVR1C	ACVR2A, ACVR2B	SMAD2/3
	GDF3	ACVR1B, ACVR1C	ACVR2A, ACVR2B	SMAD2/3
	GDF8/Myostatin	ACVR1B, ACVR1C	ACVR2A	SMAD2/3
	GDF9	ACVR1B	BMPR2	SMAD2/3
	GDF11	ACVR1B, TGFBR1	ACVR2A, ACVR2B	SMAD2/3
	GDF5	BMPR1A, BMPR1B	ACVR2A, ACVR2B, BMPR2	SMAD1/5
	GDF6	BMPR1A, BMPR1B	ACVR2A, ACVR2B, BMPR2	SMAD1/5
	GDF7	BMPR1A, BMPR1B	ACVR2A, ACVR2B, BMPR2	SMAD1/5
	GDF10	BMPR1A, BMPR1B	ACVR2A, ACVR2B, BMPR2	SMAD1/5
	INHIBIN	**-**	ACVR2A	**-**
LEFTY	LEFTY-1	**-**	**-**	**-**
	LEFTY-2	**-**	**-**	**-**
AMH		ACVR1A, BMPR1A	AMHR2	SMAD1/5

**Fig. 1:**
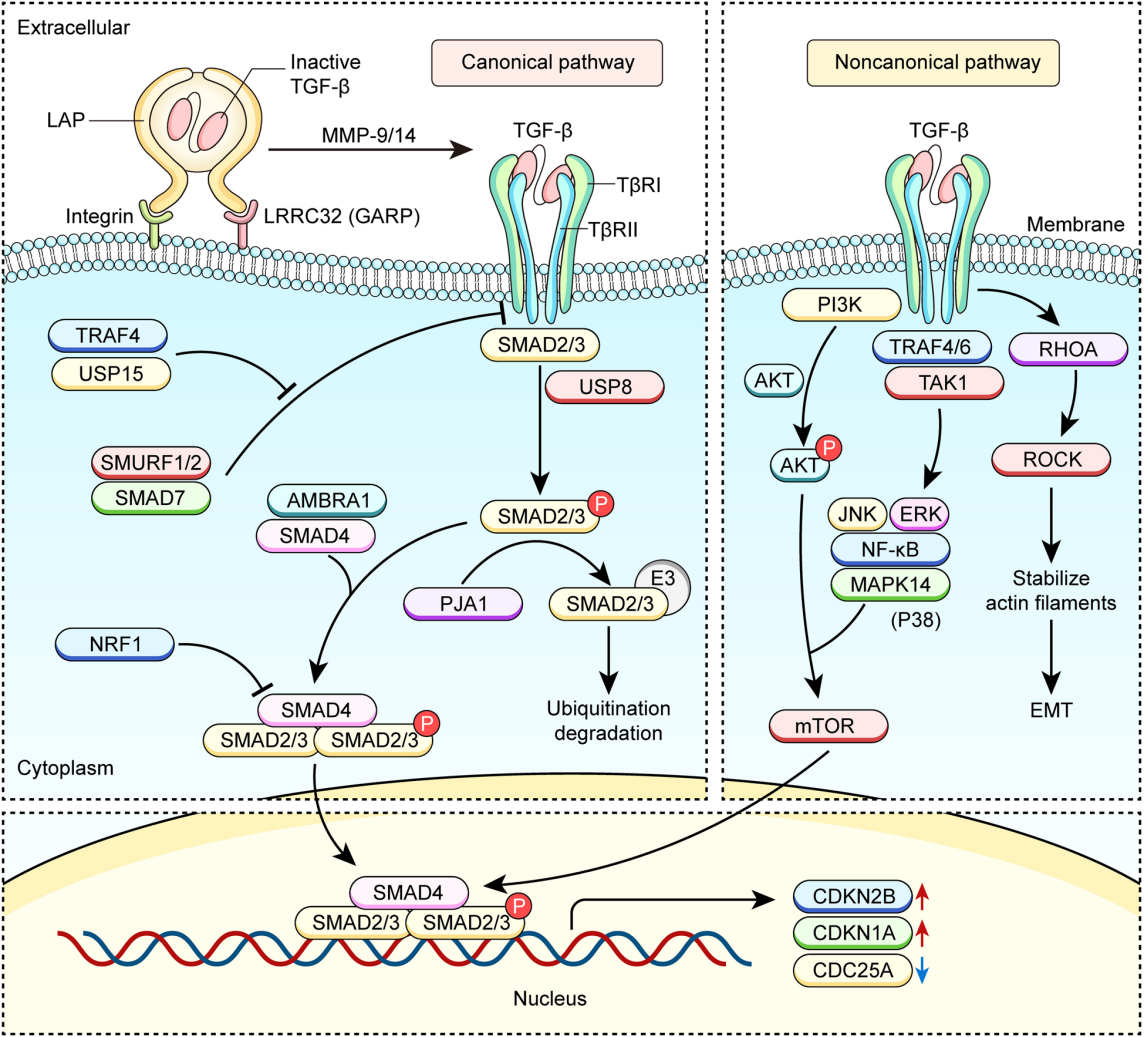
Activation of the TGF-β and TGF-β signaling pathways. TGF-β is synthesized as a disulfide-linked dimeric precursor with LAP at the N-terminal and mature TGF-β cytokine at the C-terminal. The latent TGF-β is tethered to ECM via LTBPs (not depicted here) or to cell membrane via LRRC32 (GARP). Integrins αvβ6/8, perhaps MMP-9/14 as well, can liberate and expose the receptor binding sites of active TGF-β. Upon binding to TβRI and TβRII, the TGF-β/TβRI/TβRII complex phosphorylates SMAD2/3, which subsequently translocate into the nucleus to initiate the transcription of target genes (the classical SMAD-dependent pathway). By contrast, in non-canonical SMAD-independent signaling pathways, TGF-β/TβRⅡ/TβRⅠ complex directly activates, mainly through phosphorylation, a panel of downstream signaling pathways like PI3K-AKT-mTOR, JNK and ERK, etc

The three TGF-β isoforms are produced by a variety of cell types. Each isoform is originally synthesized as a disulfide-linked dimeric precursor with the latency-associated peptide (LAP) at the N-terminal and the mature TGF-β cytokine at the C-terminal, which undergoes processing in the Golgi apparatus through cleavage by the endoprotease FURIN [7]. Each monomer of the mature TGF-β contains three internal disulfide bridges that create a compact and stable structure termed “cystine knot” featured by extended flexible loops essential for recognition by receptors and regulatory proteins [8]. Notably, following the proteolytic processing by FURIN, the TGF-β molecule is still noncovalently associated with LAP through multiple interactions, which mask TGF-β's receptor-binding domains. In most cells, this complex is further covalently linked to latent TGF-β-binding proteins (LTBPs) in extracellular matrix (ECM), or to the transmembrane leucine-rich repeat containing proteins LRRC32 (also known as glycoprotein A repetitions predominant protein GARP) and the negative regulator of reactive oxygen species NRROS (commonly known as LRRC33) on the membrane of TGF-β-producing cells [9, 10] (Fig. 1). Moreover, cells expressing integrins like αvβ6/8 can activate TGF-β1 and TGF-β3 by binding the sequence RGD (Arg-Gly-Asp) present in an exposed loop of their structures, which subsequently induces a physical force deforming the tethered latent complex to either release free active TGF-β or expose the receptor binding sites of this captive cytokine [11, 12]. In addition, LAP may present potential cleavage sites for proteases like matrix metalloproteinase-9/14 (MMP-9/14) and serine proteases (plasmin and cathepsin D) to release active TGF-β in vitro [13, 14]. After these processes, TGF-β may bind to the receptor on the cell surface to initiate downstream signal transductions, and thereby exerts the biological functions.

The TGF-β/TGF-β receptor (TβR) hetero-tetrameric complex transmits signals through both the canonical SMAD-dependent and the noncanonical SMAD-independent pathways (Fig. 1). SMAD family transcription factors (TFs) are directly phosphorylated by type I receptor kinases. Structurally, these proteins contain two conserved globular domains, the N-terminal and C-terminal regions linked by an unstructured segment [15]. The N-terminal domain binds to DNA, while the C-terminal domain mediates interactions with type I receptors, adaptor proteins, nuclear transport regulators, transcriptional cofactors (e.g., p300/CBP), chromatin-modifying proteins, and other SMADs. In the canonical pathway, TGF-β-activated type I receptors predominantly target SMAD2 and SMAD3 for phosphorylation. Subsequently, the phosphorylated SMAD2/3, together with SMAD4, enter the nucleus to initiate the transcription of target genes. By contrast, in the non-canonical pathway, the TGF-β/TβRⅡ/TβRⅠ hetero-complex directly modifies key effectors, mainly through phosphorylation, to activate the downstream signaling pathways like PI3K-AKT-mTOR, JNK, NF-ҡB, MAPK14 (P38), ERK, and RHO. The latter non-canonical signaling pathway greatly expands the involvement of TGF-β signaling in cellular events, such as proliferation, differentiation, and mitochondrial metabolism [16].

Regulation of the TGF-β signaling pathway is accomplished by a variety of molecules capable of modulating the expression and activity of SMADs and/or TβRs at multiple levels (Fig. 1) [17]. For example, SMAD7 not only recruits SMURF1/2 to the cytoplasm to promote ubiquitination-mediated degradation of TβRⅠ, but also may bind to GADD34-PP1c, which subsequently dephosphorylates and thus inactivates TβRⅠ [18]. In contrast, TNF receptor associated factor 4 (TRAF4) inhibits SMURF2 and enhances the recruitment of USP15 to TβRⅠ, ultimately stabilizing the TGF-β signaling pathway [19]. Moreover, a recent study revealed that USP8, another deubiquitinating enzyme, potentiated TGF-β signaling by deubiquitinating TβRⅡ, and accordingly, inhibiting the activity of USP8 boosted the anti-tumor immune responses via attenuating TGF-β signaling [17]. Apart from TGF-β receptors, the E3 ubiquitin ligase PJA1 inhibits TGF-β signaling by promoting the ubiquitination of phosphorylated SMAD (pSMAD) [20], whereas AMBRA1 cooperates with the CUL4-RING ubiquitin ligase complex to augment the nonproteolytic K63-linked polyubiquitination of SMAD4 and thereby enhance its transcriptional activity [21]. Besides the regulation at the protein level, the nuclear respiratory factor 1 (NRF1) binds to the promoter region of *Smad4* and represses its transcription, and thus disrupts TGF-β-SMAD4-mediated induction of CDKN2B that promotes tumor development [22].

## Contextual duality of TGF-β in tumor development and progression

### Early tumor suppression

**Fig. 2:**
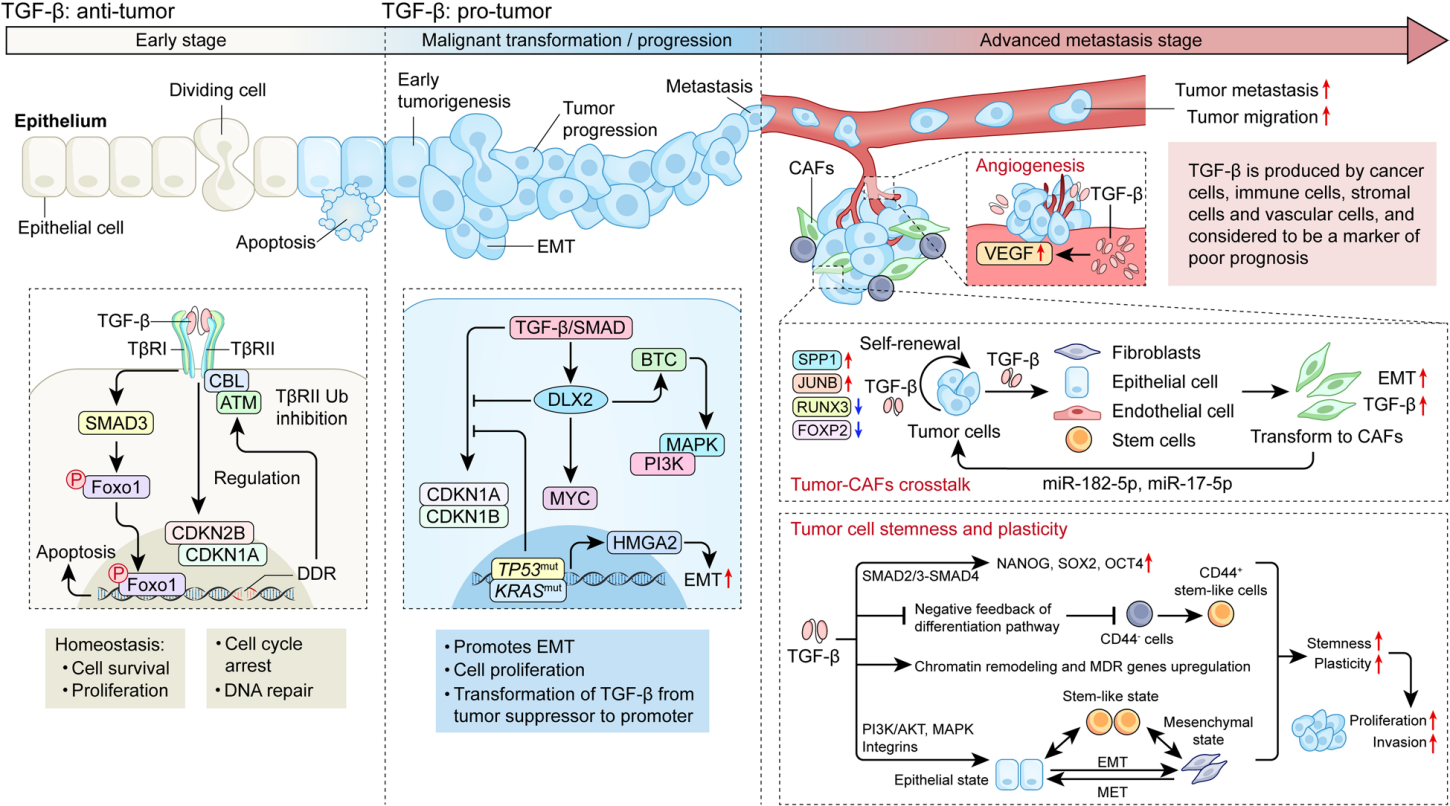
Roles of TGF-β in tumor development, progression and metastasis. During the early stages of carcinogenesis, TGF-β exerts an anti-tumor effect mainly by functioning as a cell-cycle checkpoint to regulate the survival, genome integrity and proliferation of cells, thereby repressing the malignant transformation of normal cells (*left panel*). However, mutations of *TP53* and *KRAS* and/or expression of DLX2 shift TGF-β signaling from apoptosis-induction to EMT/proliferation-promotion, switching TGF-β from tumor suppressor to promoter (*middle panel*). In the advanced tumor tissues, high levels of TGF-β, derived from multiple types of cells, facilitate the metastasis/dissemination of tumors through: 1) inducing angiogenesis via upregulating VEGF production; 2) converting the transformation of normal cells to CAFs, which promote cancer cell proliferation; and 3) promoting the stemness and plasticity of tumor cells (*right panel*). Please refer to the text for details

As a pleiotropic cytokine, TGF-β exerts potent tumor-suppressive effects during early tumorigenesis by inducing G1-S cell cycle arrest, promoting apoptosis, and maintaining genomic stability (Fig. 2). Activated TGF-β signaling mainly functions as a G1-S cell cycle checkpoint to regulate the survival and proliferation of cells through modulating the expression of CDKN2B, CDC25A, and/or CDKN1A [4]. TGF-β also regulates the apoptosis of cells through activating the downstream SMAD and/or FOXO1 signaling pathways, which represses the mitogenic TF MYC and shifts the balance between the BIM (pro-apoptotic) and BCL-2 (anti-apoptotic) [6, 23]. Together, these processes effectively restrict the transformation of normal cells to tumor cells. Moreover, TGF-β signaling participates in maintaining genomic integrity of cells in the context of DNA damage repair (DDR) [2]. Activated DDR induces the interaction among ATM, CBL, and TβRII, which subsequently prevents TβRII’s ubiquitination-dependent degradation by promoting its neddylation [24]. This process ensures a full cell-cycle arrest to allow for efficient DDR. TGF-β upregulates key components of homologous recombination (HR), such as BRCA1 and RAD51, to promote the efficient repair of double-strand breaks (DSBs). Additionally, it facilitates the recruitment of repair complexes to bulky DNA adducts (e.g., UV-induced damage), thereby participating in nucleotide excision repair (NER) [25].

### Tumor transformation induction

TGF-β transits from a tumor suppressor to a tumor promoter through multidimensional mechanisms (Fig. 2). Tumor cells tend to escape from the anti-proliferation and pro-apoptosis effects of TGF-β by deregulating the cell cycle machinery, thereby resulting in proliferative advantages and tumor progression. For example, *TP53*-mutated ovarian cancer (OC) cells, which account for up to 96% of chemotherapy-resistant, high-grade serous OC identified by The Cancer Genome Atlas (TCGA), do not respond to TGF-β-induced growth inhibition [26, 27]. Together with the *TP53* mutation, *KRAS*^*G12D*^ mutation protects intestinal tumor-derived organoid cells from TGF-β-induced growth suppression by inhibiting CDKN1A and CDKN1B expression, and promotes partial EMT *via* upregulating HMGA2, a cofactor of the SMAD complex (Fig. 2) [28].

Distal-less homeobox 2 (DLX2), induced by TGF-β/SMAD4 signaling, is a direct transcriptional repressor of TβRII. DLX2 inhibits the canonical SMAD-dependent TGF-β signaling and the expression of the cell-cycle inhibitor *CDKN1A*, while increasing the expression of the MYC protein [29]. Moreover, DLX2 functions synergistically with SMAD-independent TGF-β signaling to drive the activation of MAPK and PI3K by directly inducing the expression of the epidermal growth factor (EGF) family member betacellulin (BTC), an upstream activator of the MAPK and PI3K pathways (Fig. 2) [29]. Thus, DLX2 plays a critical role in shifting TGF-β from tumor-suppressor to tumor-promoter, and levels of DLX2 positively correlate with tumor malignancy in a variety of human cancers, including gastric adenocarcinoma, acute lymphoblastic leukemia, melanoma, glioma, breast, lung, and prostate cancer [30].

### Tumor progression and metastasis promotion

The TME is a highly heterogeneous and dynamic system mainly comprising the ECM, tumor cells, immune cells, stromal cells, and multiple surface/soluble molecules as well as plenty of metabolites [31]. As a central orchestrator in the TME, TGF-β, abundantly produced by cancer, stromal, and immune cells, is strongly associated with poor prognosis and promotes tumor progression by increasing the proliferation, EMT, angiogenesis, stemness, and cellular plasticity (Fig. 2) [32].

TGF-β stimulates cancer-associated fibroblasts (CAFs) to secrete pro-angiogenic factors (VEGFA and HGF), thereby promoting vascularization [33, 34]. Unlike normal fibroblasts, CAFs display characteristic surface markers such as α-SMA, PDGFRα, and FAP [35]. Notably, CAFs serve as both a source and an amplifier of TGF-β signaling through several positive feedback loops. First, TGF-β signaling upregulates the expression of AP-1 (JUN-FOS), which subsequently binds to TGF-β1’s promoter to promote its transcription [36]; and second, high concentrations of TGF-β in the TME may also transform epithelial, endothelial, fibroblast and mesenchymal stem cells (MSCs) into CAFs capable of producing TGF-β and MMP-2/9, which digest matrix membranes and release active TGF-β from the LAP-TGF-β dimer [37]. In addition, TGF-β signaling promotes miR-17-5p expression in CAFs, which can be delivered into colon cancer cells through exosomes to inhibit RUNX3, eventually leading to elevated TGF-β production and tumor progression [38]. Furthermore, TGF-β also downregulates FOXP2 via miR-182-5p, thereby relieving the former’s inhibitory effect on TGF-β signaling [39]. As such, TGF-β, through all these positive feedback regulations, potentiates its own signaling and production in the TME (Fig. 2).

Integrin αvβ6-TβR interactions promote tumor cell motility and invasion via the SMAD2-β-catenin signaling [40–42]. It was found that upregulated CXCR4 and TGF-β in colon cancer largely contribute to cancer metastasis and invasion. The squamous carcinoma cells (SCCs) cultured with CAFs-conditioned medium remarkably increase SPP1, JUNB and VEGFA expression, which promote the angiogenesis as well as cancer cell self-renewal and metastasis/migration (Fig. 2) [43]. Notably, about 40% of all TGF-β in peripheral blood plasma is secreted by platelets. Higher TGF-β levels were observed in OC patients with elevated platelet counts, which were correlated with higher incidence of advanced tumor stages [44], possibly owing to the fact that platelet TGF-β1 promotes the growth of primary OCs via facilitating neoangiogenesis by upregulating the expression and activity of VEGF [45]. In hepatocellular carcinoma (HCC), platelet-derived TGF-β1 promotes EMT and metastasis [46]. Moreover, expression of TGF-β1 is significantly positively correlated with the VEGF level and microvascular density in colorectal cancer (CRC) patients [47], and TGF-β signaling in the TME may promote cancer cell proliferation and angiogenesis by activating the HBEGF/IL-1β/EREG pathway [48]. In addition, TGF-β signaling upregulates a panel of factors, including ANKRD1, CCBE1, FSTL3, PLAU, THBS1 and ITGB3, that have been implicated in the migration and angiogenesis of multiple types of cancers, and thereby represent poor-prognosis markers in patients with head and neck squamous cell carcinoma (HNSCC). Consequently, blockade of TGF-β signaling suppresses tumor growth and angiogenesis in a subcutaneous xenograft model of oral cancer cells with high TGF-β expression [49].

Moreover, TGF-β acts as a critical regulator in modulating carcinoma cell progression and metastasis partially by inducing EMT (Fig. 2) [50]. TGF-β induces EMT by upregulating TFs (e.g., SNAI1, ZEB1, and TWIST) via SMAD3 and STAT3 cooperation while suppressing epithelial markers like E-cadherin, leading to an enhanced mesenchymal phenotype (Vimentin). Moreover, TGF-β further stabilizes the EMT phenotype through non-coding RNAs and epigenetic mechanisms [51]. EMT manifests as a continuous progression where cells transition from a fully epithelial phenotype to a fully mesenchymal phenotype through various intermediate hybrid states. Interestingly, recent studies have demonstrated that TGF-β can induce partial EMT (pEMT) in a variety of epithelial cells, and this intermediate state is associated with cancer cell stemness, oncogenicity, and metastasis [52–55]. In HNSCC, elevated TGF-β expression is positively correlated with the proportion of pEMT, and a higher pEMT score is associated with advanced tumor grade and increased incidence of lymph node metastasis [56]. Notably, TGF-β promotes chemoresistance by inducing EMT, for instance, by inducing a reversible G0 quiescent state in cancer cells to evade drug-induced killing [57–59], thereby conferring multidrug resistance (MDR) through metabolic reprogramming [52, 60] and enhancing cancer stem cell properties that further support cell survival and foster resistance development [61, 62].

In addition, TGF-β sustains the stemness and plasticity of cancer cells through transcriptional regulation, differentiation blockade, and epigenetic reprogramming [63]. Mechanistically, it directly activates stemness factors (*SOX2*, *NANOG*, and *OCT4*) *via* the TGF-β/SMAD2/3-SMAD4 axis, induces the conversion of CD44^-^ cells into CD44⁺ stem-like cells by inhibiting the negative feedback of differentiation pathways (such as WNT/β-catenin), remodels chromatin through H3K27me3 demethylation, and upregulates MDR genes like *ABCG2* [6, 63, 64]. This enables cancer stem cells (CSCs) to adapt and survive under microenvironmental stresses such as hypoxia and nutrient deprivation through EMT-MET (mesenchymal epithelial transition) processes, ultimately driving tumor proliferation, recurrence, and therapeutic resistance [40]. Moreover, TGF-β regulates the expression of ATXN1 (SCA-1) and the plasticity of murine mammary epithelial stem cells, promotes the development of CSC properties and drives tumor formation and progression [65]. By synergizing with signaling cascades such as PI3K/AKT, MAPK, and integrins, TGF-β further enhances the cellular plasticity, which allows tumor cells to dynamically transit among epithelial, mesenchymal, and stem-like states, thereby enabling them to better adapt to the constantly changing microenvironment and evade therapeutic interventions (Fig. 2) [66–69]. These features not only fuel tumor cell proliferation and invasion but also confer immune evasion, as shown in triple-negative breast cancer (TNBC) where αvβ6-TGF-β signaling induces SOX4 expression to suppress antigen presentation [70]. In gliomas and non-small cell lung cancer (NSCLC), TGF-β additionally silences the transcription of the tumor suppressor *MST1* through the epigenetic regulator DNMT1 (DNA methyltransferase 1), further reinforcing the plasticity and malignant progression [71–73].

Notably, TGF-β can decouple proliferation from motility. Yutaro Tsubakihara et al. disclosed a high tumor-initiating capacity but limited metastatic potential of TGF-β-treated mammospheres [74]. In contrast, TGF-β confers higher motility and metastatic ability to oral cancer cells arrested in the G1 phase by upregulating the expression of KRTAP2-3 in a SMAD4-dependent manner, which orchestrates cancer cell proliferation and migration by inducing EMT through *ZBED2* and *ENC1* [75]. Consequently, the TGF-β/KRTAP2-3 axis is associated with poor prognosis in patients with HNSCC, and motile cancer cells arrested in the G1 phase may represent a target to suppress metastasis in such patients [75].

### Genomic alterations in TGF-β pathway components in cancers

Genomic alterations in core components of the TGF-β signaling pathway, including receptors, SMAD transducers, and regulatory genes, are prevalent across human cancers and profoundly influence the pathway’s signaling output, often shifting the balance from tumor suppression to oncogenesis.

Approximately 39% of tumors analyzed in the TCGA pan-cancer studies harbor genetic alterations in TGF-β pathway genes, with particularly high frequencies in gastrointestinal malignancies [76]. These alterations disrupt canonical TGF-β signaling, leading to loss of growth control and apoptosis, while enabling pro-tumorigenic processes such as EMT and metastatic dissemination [77]. Key among these are inactivating mutations in *TGFBR2* and *SMAD4*, which are linked to aggressive disease progression and poor prognosis in CRC and pancreatic ductal adenocarcinoma (PDAC) patients. For example, *SMAD4* loss is correlated with enhanced local invasion, metastasis, and shortened survival [78]. Additionally, deficient TGF-β signaling promotes genomic instability through upregulation of error-prone alternative end-joining (alt-EJ) DNA repair, which increases the sensitivity to genotoxic agents but also accelerates mutagenesis and tumor evolution.

Epigenetic silencing also contributes to pathway dysfunction. In HNSCC patients, promoter hypermethylation of key genes, including *TGFBR1, TGFBR2, SMAD4, CDKN2A, and DPAK1*, abrogates TGF-β-mediated growth regulation, fostering uncontrolled survival and proliferation of cancer cells [79].

Thus, genomic and epigenetic inactivation of TGF-β pathway components represents a common mechanism enabling immune evasion, metastasis, and therapeutic resistance of human cancer cells, underscoring its role as a critical tumor suppressor whose loss defines a more aggressive disease subset.

## TGF-β as a master regulator of metastatic cascades

### Orchestrating local invasion

**Fig. 3:**
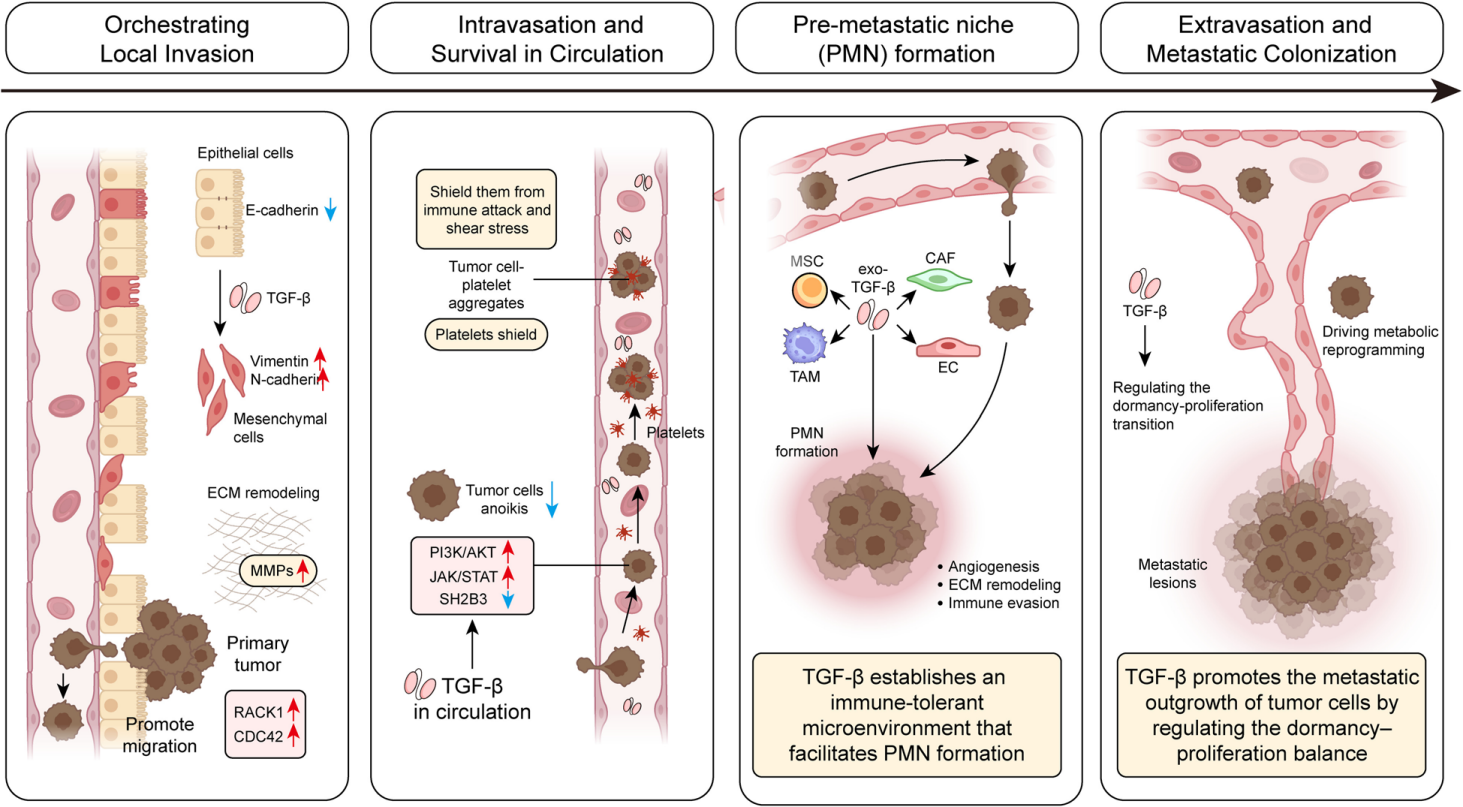
TGF-β regulates the metastatic cascades in tumor cells. TGF-β promotes the local invasion of tumor cells by inducing EMT, ECM remodeling, MMP production as well as motility of tumor cells. After detachment from the ECM and entering the circulation, TGF-β signaling confers anoikis resistance to tumor cells through modulating PI3K/AKT, JAK/STAT and SH2B2 pathways. Moreover, tumor cell-platelet aggregates promote the survival of tumor cells by shielding them from immune attack and shear stress. In potential metastatic organs, tumor-derived exosomes loaded with TGF-β (exo-TGF-β) facilitate the pre-metastatic niche (PMN) formation by inducing angiogenesis, ECM remodeling, and immune evasion. Finally, after extravasation, TGF-β fine-tunes the dormancy-proliferation transition of tumor cells via cell-cycle regulation and metabolic reprogramming, eventually fostering their metastatic outgrowth in distant secondary sites

Tumor metastasis is the leading cause of cancer mortality, with local invasion as the first step. Tumor cells detach from the primary site and penetrate surrounding tissues, and the process of EMT allows epithelial cells to acquire mesenchymal traits, thereby enhancing migratory and invasive capabilities [80, 81]. TGF-β is a key cytokine that promotes EMT and facilitates local tumor invasion (Fig. 3).

As discussed above, TGF-β induces TFs like *ZEB1*, *SNAI1*, and *ZEB2 *via the canonical SMAD2/3-SMAD4 signaling, which subsequently promotes EMT by downregulating epithelial markers (E-cadherin) while upregulating mesenchymal markers (Vimentin and N-cadherin), thereby promoting cell migration and invasion [82–84]. For example, ERBB4 activates *SNAI1 *via SMAD4 phosphorylation, promoting the migration of NSCLC cells [85]. In HCC, MICAL1 potentiates TGF-β signaling and thus promotes metastasis by stabilizing TβRI and increasing SMAD2/3 phosphorylation [86]. The effect of TGF-β on EMT is further amplified by non-canonical pathways like MAPK/ERK and PI3K/AKT, and reinforced through regulatory mechanisms such as δEF1/ZEB1-mediated suppression of epithelial splicing regulatory proteins ESRPs, which shifts splicing patterns toward mesenchymal isoforms [84, 87, 88]. Concurrently, TGF-β systematically remodels the ECM by upregulating MMPs. It enhances MMP-2 and MMP-9 expressions through SMAD-dependent transcription, ROS-NF-κB activation, and MAPK/PI3K pathways [89–91]. A positive feedback loop involving MMP14 and WNT5A further sustains MMP production and EMT in prostate cancer [92]. Epigenetic mechanisms also contribute to this process as seen via SOX9-mediated transactivation of MMP-10 [93]. As a consequence, these MMPs degrade collagen and other ECM components, thereby breaking down physical barriers to tumor invasion. In melanoma, MMP-14 inhibition reduces collagen cleavage and restores drug sensitivity [94]. TGF-β also alters ECM composition by inducing collagen synthesis and disrupting the MMP/TIMP (MMP inhibitors) balance, fostering a pro-invasive microenvironment, as demonstrated in glioma [95].

Finally, TGF-β promotes migration by driving cytoskeletal dynamics. It activates RHOA-ROCK to stabilize actin filaments and promotes RAC1 and CDC42, thus enhancing actomyosin contractility and facilitating lamellipodia and filopodia formation, respectively [96, 97]. The MAPK pathway synergizes with these processes by promoting stress fiber assembly and actin-related protein 2/3 (ARP2/3)-mediated actin branching, ensuring coordinated cell movement [98].

### Intravasation and survival in circulation

Anoikis, a specialized form of programmed cell death triggered by the loss of ECM attachment, represents a major barrier to hematogenous dissemination, as metastatic tumor cells must acquire resistance to anoikis to survive within the circulatory system [99]. TGF-β signaling confers anoikis resistance to tumor cells through multiple pathways **(**Fig. 3**)**. For instance, through SMAD-dependent and -independent pathways (e.g., JAK/STAT, PI3K/AKT, and MAPK), TGF-β induces EMT, thereby facilitating the detachment from primary lesions while suppressing apoptosis simultaneously [100, 101]. Moreover, TGF-β1 derepresses the inhibitory effect of SH2B3 on JAK2/STAT3 and PI3K/AKT signaling cascades by downregulating its expression, leading to enhanced anoikis resistance, EMT, and metastatic potential of lung cancer cells [102]. TGF-β signaling drives GLI2 activation in CRC cells, which reinforces anoikis resistance and immune evasion via the non-canonical Hedgehog signaling [103]. In TNBC, IL-17A cooperates with TGF-β signaling to upregulate the anti-apoptotic BCL-2 family proteins, thus preventing anoikis while augmenting the migratory capacity [104]. As such, targeting GLI2 and IL-17A in vivo may hold potential in cancer treatment by reducing anoikis resistance, immune evasion, and metastatic dissemination.

Apart from serving as an important source of TGF-β [46, 88], platelets rapidly cloak circulating tumor cells, forming tumor cell-platelet aggregates (TCIPA) that shield them from immune attack and shear stress **(**Fig. 3**)** [105]. TCIPA correlates with increased metastasis alongside poor prognosis across pancreatic, breast, and OC cancers in the clinic, and preclinical studies have shown that platelet depletion or TCIPA inhibition markedly reduces metastatic colonization [106, 107]. Accordingly, emerging nanomedicine strategies targeting platelet receptors or modulating platelet-leukocyte interactions have demonstrated potential to disrupt platelet-mediated tumor survival and suppress immune evasion [108].

### Pre-metastatic niche formation

The pre-metastatic niche (PMN), a microenvironment established by the primary tumor in potential metastatic organs, plays a crucial role in the process of tumor metastasis by providing suitable conditions for circulating tumor cells to colonize, survive, and proliferate [109]. A key mechanism of this remote regulation is through tumor-derived exosomes, which serve as stable carriers for TGF-β **(**Fig. 3**)** [110]. For example, in TNBC, acetylation of LAP-TGF-β1 by TIP60 enables HSP90A-assisted loading of TGF-β into exosomes, and thus inhibition of this pathway reduces exosomal TGF-β by ~ 70% and suppresses lung metastasis [111]. Beyond TGF-β delivery, exosomes themselves contribute to PMN formation as well. In renal cell carcinoma (RCC), CD105⁺ CSCs-derived exosomes promote angiogenesis, thereby facilitating the formation of pulmonary PMN and increasing the metastatic burden by 2–threefold [112].

Within target organs, TGF-β establishes an immune-tolerant microenvironment that facilitates PMN formation [113]. Moreover, TGF-β cooperates with stromal cell components such as CAFs, endothelial cells (ECs), MSCs, and tumor-associated macrophages (TAMs) to enhance angiogenesis, ECM remodeling, and immune evasion [114–116], all of which contribute to the establishment of PMN.

In summary, targeting TGF-β to inhibit PMN formation has the potential to significantly reduce metastasis risk while augmenting the efficacy of immunotherapy, positioning it as a promising therapeutic target for cancer intervention [109].

### Extravasation and metastatic colonization

After intravasation and circulation, disseminated tumor cells that reach secondary sites often enter a dormant state to evade immune surveillance and microenvironmental stresses [117]. The TGF-β signaling pathway plays a pivotal role in regulating this dormancy-proliferation balance **(**Fig. 3). Through p38 MAPK activation, TGF-β upregulates the cell cycle inhibitor CDKN1B, enforcing a G1 arrest that enables tumor cells to persist in a quiescent state under adverse conditions [118, 119]. Moreover, TGF-β drives metabolic reprogramming that supports long-term survival and metastatic outgrowth of tumor cells. By enhancing glycolysis via upregulation of glycolytic enzymes and simultaneously suppressing mitochondrial oxidative phosphorylation (OXPHOS) by downregulating NDUFB8 and UQCRC2, TGF-β promotes lactate accumulation and extracellular acidification, thereby sustaining energy production and fostering an immunosuppressive niche [40, 120, 121]. In addition, TGF-β fine-tunes key metabolic pathways involved in the synthesis and metabolism of macromolecules such as amino acids, nucleotides, and lipids [122]. TGF-β upregulates glutamine transporter SLC7A5 and glutaminase GLS1, boosting glutamine uptake and catabolism to provide nitrogen for nucleotide and amino acid synthesis [123–126]. TGF-β2 promotes lipid droplet formation and fatty acid utilization, both of which provide energy reserves essential for colonization in metabolically challenging microenvironments. Importantly, acidosis-induced activation of TGF-β2 enhances pEMT, linking metabolic adaptation with cellular plasticity to reinforce metastatic potential [127]. Through these mechanisms, TGF-β controls the dormancy-proliferation switch of tumor cells in the distant microenvironment by inducing metabolic transition and adaptation.

## TGF-β as a potent immune checkpoint in the TME

**Fig. 4:**
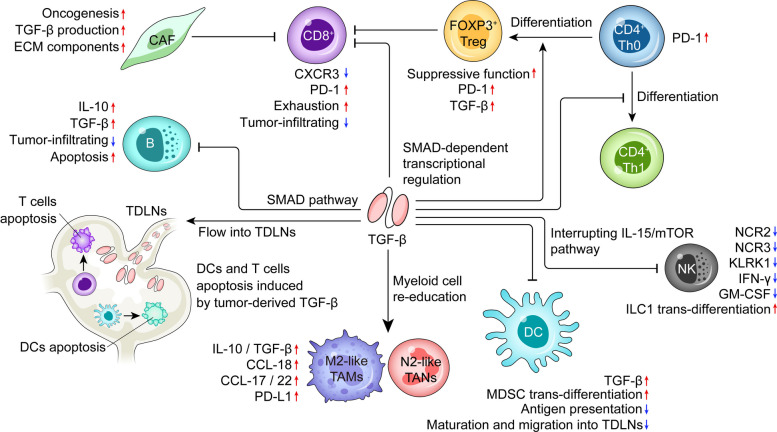
TGF-β dampens the anti-tumor immune responses by targeting multiple cell types. TGF-β may flow into the tumor draining lymph nodes (TDLNs) to induce the apoptosis of both DCs and T cells, thereby inhibiting the priming of anti-tumor T-cell responses in TDLNs. In tumor tissues, TGF-β dampens the anti-tumor immunity mainly by: 1) inhibiting tumor-eradicating ability of CD8^+^ T cells and NK cells; 2) inducing the differentiation of TAMs/TANs towards M2/N2-like TAMs/TANs with enhanced immunosuppressive functions, respectively; 3) suppressing the infiltration and antigen-presenting ability of DCs and B cells; 4) impeding the differentiation of Th1 cells while promoting the generation and suppressive function of FOXP3^+^ Tregs; and 5) activating and expanding CAFs to produce excessive amounts of ECM components, thereby creating an exclusionary TME

In healthy individuals, the development and growth of tumor cells is strictly controlled by cytotoxic innate and adaptive immune cells. Notably, apart from the direct effect on tumor cells, TGF-β promotes the growth and metastasis of tumor cells indirectly by dampening the anti-tumor immune responses through multiple mechanisms (Fig. 4).

### Direct inhibition of effector immune cells

Accumulation of T cells in the tumor is vital for effective anti-tumor response. For CD8^+^ T cells that may differentiate into cytotoxic T lymphocytes (CTLs) upon stimulation, TGF-β impairs their activity by downregulating key effector molecules, including perforin, granzymes A/B, FAS ligand, and IFN-γ. At the molecular level, SMAD proteins and the TF ATF1 directly interact with the promoter regions of *GZMB* and *IFNG* genes, leading to transcriptional inhibition upon TGF-β pathway activation. Consequently, diminished IFN-γ production is linked to decreased levels of TBX21 (T-BET), a critical regulator of Th1 responses [128]. By binding and trafficking towards its IFN-γ-inducible ligands CXCL-9/10/11, chemokine receptor CXCR3 promotes the infiltration of both endogenous and adoptively-transferred anti-tumor T cells [129]. In the TME, TGF-β represses the transcription of CXCR3 in CD8^+^ T cells, and conversely, CD8α-specific deletion of *Tgfbr1* enhances CXCR3 expression on CD8^+^ T cells and thereby facilitates their CXCR3-dependent migration into tumors [130]. Intriguingly, it has been reported that TGF-β induces a subset of CD103^+^CD8^+^ T cells in the TME with augmented CXCL-13-secreting abilities, which correlates with B-cell recruitment, formation of tertiary lymphoid structures (TLSs), and neoantigen burden in six cohorts of human tumors. As such, TGF-β may play a noncanonical role in coordinating immune responses against human tumors in some circumstances as well [131]. In addition, studies demonstrate that TGF-β upregulates PD-1 expression via SMAD3-dependent transcriptional activation during antigen stimulation, suppressing anti-tumor responses in both cultured T cells and tumor-infiltrating lymphocytes (Fig. 4) [132]. Consequently, combining systemic TGF-β neutralization using anti-TGF-β antibodies with DNA vaccination or IL-2 supplementation significantly increases tumor infiltration and expands populations of tumor-reactive CD8^+^ T cells [133, 134].

NK cells are able to trigger target cell death, for instance tumor cells, both by releasing cytotoxic granules containing granzymes/perforins and through death receptor-mediated pathways without prior sensitization [135]. Moreover, they also play immunomodulatory functions by secreting chemokines and cytokines, such as CCL-5 and IFN-γ. TGF-β/SMAD3 signaling significantly mitigates FCGR3A (CD16)-induced IFN-γ expression via suppressing TBX21 [136]. TGF-β downregulates surface expressions of NCR3 and KLRK1, thereby inhibiting NK-mediated cytotoxicity against tumor cells (Fig. 4) [137, 138]. TGF-β impairs the expression of KLRK1 via multiple mechanisms, including upregulating miR-1245 and downregulating HCST (DAP10) [137]. Meanwhile, TGF-β upregulates miR-183 in human lung cancer cells, which represses their surface MICA/B levels and thereby circumvents the detection by KLRK1 on both NK and CD8^+^ T cells [139]. TGF-β-induced miR-183 also reduces TYROBP (DAP12) expression in tumor-associated NK cells, attenuating their tumor cytolytic ability by reducing surface KIR2DS4 and NCR2 [140]. In addition, constitutive TGF-β signaling not only arrests NK cell development but also blunts their ability to limit tumor metastasis in vivo through blocking IL-15-induced mTOR activity [141]. TGF-β1 also represses GM-CSF production of NK cells via SMAD3, which may boost the anti-lung carcinoma immune responses by augmenting the function of DCs, macrophages, T cells as well as neutrophils [142]. Furthermore, TGF-β may promote the immune invasion of tumor cells by inducing the trans-differentiation of NK cells into non-cytotoxic ILC1 cells as well [143, 144].

In line with murine studies, TGF-β1 downregulates CD16 expression on NK cells in human bladder cancers likely via SMAD2/3, leading to diminished antibody-dependent cellular cytotoxicity (ADCC) against tumor cells [145]. Together with IL-15, TGF-β1 polarizes peripheral blood NK cells into an inhibitory NK-like CD103^+^CD49a^+^ subset with high expression of TNFRSF18 (GITR) and CD101 [146]. TGF-β also limits metabolic changes that accompany cell activation. Study shows that TGF-β suppresses IL-2-stimulated mitochondrial metabolism in human NK cells, reducing both OXPHOS and maximal respiratory capacity [147].

### Induction and recruitment of immunosuppressive cells

TGF-β not only induces the development/differentiation of CD4^+^CD25^+^FOXP3^+^ regulatory T cells (Tregs), but also augments their suppressive function (Fig. 4) [148]. It is thus conceivable that the abundance of TGF-β in the TME would greatly elevate the number and function of Tregs that dampen the anti-tumor immunity of T cells either directly (for instance, as an IL-2 sink) or indirectly by repressing the function of antigen-presenting cells (APCs) via CTLA-4 and LAG3 [149]. Notably, Tregs, either recruited by tumor cells via the CCL-5/CCR5 axis from the periphery or locally converted from conventional T cells (Tconv) by TGF-β in the TME, are capable of producing TGF-β as well, forming a vicious feedback cycle that aggravates immunosuppression and tumor progression [150]. Hence, high numbers of Tregs in tumor tissues are often associated with poor prognosis in cancer patients. Besides increased numbers, tumor-infiltrating Tregs, compared with those in lymphoid/non-lymphoid tissues or blood, express higher levels of cell surface molecules associated with T-cell activation and immune suppression, such as CD25, CTLA-4, PD-1, LAG3, TIGIT, ICOS, ENTPD1 (CD39), as well as a panel of TNF receptor superfamily members like TNFRSF9 (4-1BB), TNFRSF4 (OX-40), and TNFRSF18 (GITR), implying that they possess enhanced suppressive ability [151]. Akin to TNBC cells, TGF-β upregulates the TF SOX4 in Tregs as well, which, in turn, promotes CD39 expression, thereby conferring a stronger proliferative and suppressive capability to Tregs in solid tumors [152, 153]. By contrast, CD39-expressing Tconv in tumor tissues exhibit an exhausted signature [154].

TGF-β signaling also has dramatic effects on myeloid cells. In vitro, incubation of tissue macrophages with TGF-β inhibits expression of multiple characteristic genes of inflammatory macrophages, including TNF-α, IL-12, and inducible nitric oxide synthase (iNOS). In parallel, TGF-β induces expression of a suite of genes, including arginase 1 and IL-10 which are characteristic of TAMs. Mice lacking TβRII in myeloid cells demonstrate increased anti-tumor immunity, decreased tumor growth and metastasis [155, 156]. Although the mechanisms underlying these events differ among different tumor models, the decrease in metastases appears to be explained by increased pro-inflammatory cytokine productions of macrophages that are unable to respond to TGF-β. The persistent stimulation of myelopoiesis that accompanies chronic infection, inflammation, and cancer is associated with the emergence of myeloid-derived suppressor cells (MDSCs) bearing TGF-β-dependent immunosuppressive ability. MDSCs, either aberrantly generated within the TME or originated in the bone marrow followed by migration into the malignant sites, create an immunosuppressive milieu and promote immune escape of tumor cells [157, 158]. MDSCs are primarily classified into granulocytic/polymorphonuclear (G/PMN-MDSC) and monocytic (M-MDSC) subsets, distinguished by their phenotypic and morphological features. In tumor tissues, large numbers of TAMs and MDSCs, mainly composed of tumor-associated neutrophils (TANs), significantly impede the effective anti-tumor immune responses via multiple mechanisms, such as competing with T cells for nutrients and producing metabolites [31, 159].

TGF-β skews the polarization of both TAMs and TANs towards an M2/N2-like phenotype featured by the production of immunosuppressive cytokines (IL-10 and TGF-β, etc.) and chemokines that promote tumor-infiltration/metastasis (CCL-18, etc.) or recruit Tregs (CCL-17/22, etc.) into the TME (Fig. 4). In NSCLC, SMAD3 activation in TANs drives the N2-like polarization [160]. In macrophages, TGF-β or MSC-conditioned medium suppresses pro-inflammatory cytokines (TNF-α and IL-6) while upregulating M2 markers (ARG-1 and IL-10), a process mediated by AKT-dependent inhibition of FOXO1 nuclear translocation [161]. Additionally, stromal-derived periostin (POSTN), highly expressed in aggressive OC, activates integrins (αvβ3/β5) to trigger NF-κB signaling, leading to M2 macrophage recruitment and polarization. Thus, blocking POSTN significantly reduces TAMs infiltration, CAFs activation, and tumor metastasis in preclinical OC models [162]. Furthermore, high expression of YAP and STAT3 in tumor cells facilitates the transcription of *VEGF*, which enhances PD-L1 expression on M2-like TAMs via the VEGFR1-TGF-β signaling pathway [163].

### Impairment of antigen presentation

Recent studies highlight that TGF-β functions as a master regulator of dendritic cells (DCs) dysfunction in cancers by influencing their maturation, antigen presentation, and immunomodulatory properties [164]. Mechanistically, TGF-β impairs the antigen-presenting ability of DCs through downregulating major histocompatibility complex (MHC) molecules and costimulatory molecules (CD80 and CD40) and inhibiting their migration into draining lymph nodes (DLNs) [165–167]. Moreover, tumor-derived TGF-β1 may flow into tumor DLNs (TDLNs) to induce immunosuppression before the arrival of tumor cells by promoting the apoptosis of both DCs and T cells, thereby facilitating tumor metastasis within these nodes [168]. Furthermore, TGF-β upregulates PD-L1 expression on DCs, which promotes T cell apoptosis/exhaustion and expands the pool of Tregs, thereby suppressing T cell-mediated cytotoxicity against tumor cells (Fig. 4) [169, 170]. These mechanisms collectively contribute to immune dysfunction and ultimately lead to the failure of anti-tumor immune responses. Clinically, elevated TGF-β1 levels correlate with impaired DC maturation and tissue infiltration. In gastric cancer and CRC with liver metastasis, cytoplasmic TGF-β1 in tumor cells is linked to reduced infiltration of CD1a^+^ and CD83^+^ DCs within tumor sites [171]. Moreover, tumor-educated DCs produce TGF-β as well, which skews naive CD4^+^ T cells toward immunosuppressive FOXP3^+^ Tregs rather than Th1 effector cells in lung carcinoma models [172].

### Establishment of an exclusionary TME

A key mechanism by which TGF-β drives tumor progression is through the establishment of an exclusionary TME, characterized by physical and functional barriers that prevent the infiltration of immune cells to mount an effective local anti-tumor immune response. At the structural level, TGF-β activates and expands CAFs capable of secreting excessive amounts of ECM components such as collagens, causing ECM stiffening that physically impedes the infiltration of immune cells and the immune-tumor cell interactions (Fig. 4) [172–174]. Moreover, TGF-β synergizes with VEGF to promote aberrant angiogenesis, which exacerbates stromal remodeling and immune exclusion. Preclinical evidence demonstrates that bispecific blockade of TGF-β and VEGF (e.g., Y332D) restores CD8^+^ T cell infiltration and enhances systemic anti-tumor immunity in combination with radiotherapy [175]. At the functional level, CAFs secrete large quantities of growth factors, cytokines and chemokines (TGF-β, IL-6 and CCL2, etc.) to recruit immune cells, especially immunosuppressive cells, into the tumor stroma to promote immune evasion [176]. For example, *HIC1*-null prostate cancer cell-derived TGF-β converts fibroblasts to CAFs, which not only secrete high levels of CXCL-12 but also interact with M2-type TAMs to induce EMT, further complicating the immune exclusionary nature of the TME [177].

### Interplay with other immune checkpoint molecules

Beyond Tregs, TGF-β impairs the anti-tumor ability of T cells via potentiating the PD-L1/PD-1 signaling pathway in the TME. PD-1 represents an important checkpoint receptor on the surface of activated T cells. Upon interacting with its ligand PD-L1 expressed on a variety of cells in the TME like tumor cells, PD-1 negatively regulates the effector function of T cells through outside-in signals, resulting in exhaustion and attenuated anti-tumor immune responses. TGF-β1 directly potentiates antigen-induced PD-1 expression on T cells in a SMAD3-dependent but SMAD2-independent fashion both in vitro and in vivo [36, 178]. In colon cancer models, TGF-β signaling upregulates PD-L1 expression on tumor cells across various malignancies. TGF-β inhibition reversed PD-L1 induction and reduced metastatic burden by ~ 70% in murine CRC models [179]. Similarly, in HCC, TGF-β1 induces SOX18 expression, directly activating *PD-L1* and *CXCL-12* transcription to suppress CD8^+^ T cells while promoting tumor metastasis [180]. In gastric cancer, CAF-derived lysyl oxidase (LOX) enhances PD-L1 expression via histone lactylation and concurrently activates TGF-β/IGF1 signaling to drive EMT, jointly reinforcing immune evasion and invasiveness of tumor cells [181].

TGF-β also synergizes with CTLA-4, which inhibits T cell priming by outcompeting CD28 for B7 ligands, to promote the differentiation of CTLA-4^high^-expressing Tregs, thereby enriching the CTLA-4^high^ suppressive Treg population in tumors [170]. In prostate cancer, antigen-specific CD8^+^ Tregs exert suppression through CTLA-4 expression and IL-35 secretion, highlighting novel avenues for combinatorial blockade [179, 182]. Expanding beyond canonical checkpoints, dual inhibition of VISTA and CTLA-4 in breast cancer models reversed T cell exhaustion, restored CD8^+^ T infiltration, and achieved superior tumor control (78% growth suppression) as compared with each monotherapy [183].

## Types and challenges of TGF-β-targeted therapy

### Types of TGF-β targeted inhibitors

**Fig. 5:**
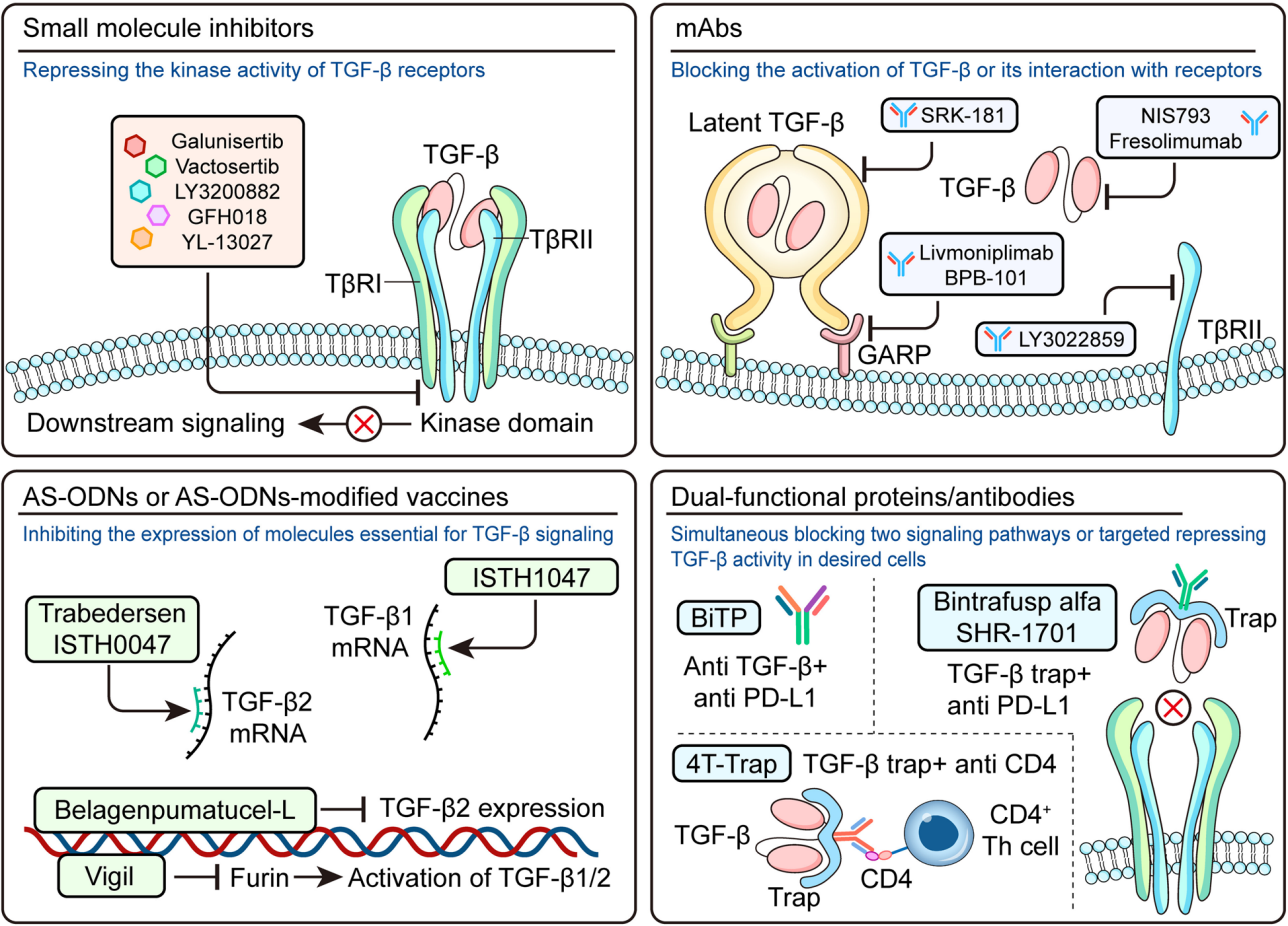
Types and action mechanisms TGF-β-signaling targeting agents. Small molecule inhibitors suppress the kinase activity of TGF-β receptors, thereby disrupting the downstream signaling transduction. mAbs bind to the latent/active TGF-β, TβRII or GARP, and thus inhibit the activation of TGF-β or its interaction with cognate receptors. AS-ODNs or AS-ODNs-modified vaccines directly repress the expression of TGF-β or key molecules, such as FURIN, essential for the activation of TGF-β. Dual-functional proteins containing the ectodomain of TGF-β receptor fused to antibodies against immune checkpoint molecules (i.e., PD-L1) or cell-lineage markers (like CD4), and hybrid bispecific antibodies targeting both TGF-β and PD-L1, are generated to increase the efficacy of these therapeutic agents by simultaneous blocking two signaling pathways or selectively repressing TGF-β activity in desired cells

The significant promoting effects of TGF-β in various tumor development and progression, as summarized in Table 2, have inspired researchers and pharmaceutical companies to develop diverse inhibitors targeting the synthesis, activation and signal transduction molecules along the TGF-β signaling pathway (Fig. 5), some of which have shown varying potential in preclinical and clinical trials for tumor therapy. Thus, this section provides an overview of major TGF-β inhibitor types, their mechanisms of action, preclinical evidence, and clinical progress. We will highlight the unresolved challenges in translating these agents to the clinic as well.

**Table 2:** Roles of TGF-β in various cancers

Tumors	Roles of TGF-β	References
Glioma	Promotes tumor growth; maintains self-renewal of glioma initiating stem cells; inhibits anti-tumor immunity; and facilitates tumor invasion via VEGF and MMPs	[191]
Brain Aneurysm	Promotes aneurysm progression and elevates the rupture risk by increasing macrophages infiltration and MMPs production; and disrupts the vascular wall structure by inducing smooth muscle cell apoptosis	[192]
Oral Cancer	Initially acts as a tumor suppressor by inducing cell cycle arrest and promoting apoptosis; and promotes tumor invasion, metastasis and immune evasion by inducing EMT and immune suppression in advanced stages	[193]
Thyroid Cancer	Promotes tumor invasion and metastasis by inducing EMT; and induces tumor recurrence and drug resistance by maintaining and expanding cancer stem cells	[194]
Esophageal Cancer	Promotes metastasis by enhancing the migratory and invasive capabilities of cancer cells	[195]
HNSCC	Acts as a tumor suppressor by inhibiting cell proliferation and inducing apoptosis during early carcinogenesis, while promoting tumor progression by shaping an immune suppressive TME in later stages	[196]
Lung Cancer	Promotes tumor progression via inducting EMT, inhibiting the anti-tumor immunity, facilitating angiogenesis and remodeling the ECM	[197]
Breast Cancer	Acts as a tumor suppressor by inhibiting cell proliferation, promoting differentiation, and inducing apoptosis in the early stage; and promotes metastasis, angiogenesis and EMT, while inhibiting the anti-tumor immune responses in advanced cancer stages	[198, 199]
Gastric Cancer	Fosters a microenvironment conducive to tumor progression and immune escape by inducing EMT and Tregs, promoting the differentiation of CAFs, and stimulating angiogenesis	[195, 200]
Liver Cancer	Enhances the invasive capacity of cancer cells by promoting EMT and drives malignant tumor progression by facilitating vascular invasion and metastasis during liver cirrhosis; and promotes the initiation and progression of liver cancer by driving liver fibrosis via inducing excessive deposition of ECM	[201, 202]
Pancreatic Cancer	Initially inhibits tumor formation by inducing cell cycle arrest and apoptosis; and later promotes tumor invasion and metastasis by inducing EMT and activating pancreatic stellate cells (PSCs)	[203, 204]
CRC	Promotes cancer cell invasion and metastasis by inducing EMT, stimulating angiogenesis and creating an immunosuppressive and pro-metastatic TME	[205]
Renal Carcinoma	Favors cancer progression by inducing EMT and inhibiting anti-tumor immunity	[206]
Bladder Cancer	Drives tumor invasion, progression, and metastasis by inducing EMT, angiogenesis and immunosuppression	[207]
Cervical Cancer	Promotes the survival and proliferation of cancer cells; and enhances tumorigenesis and metastatic dissemination by activating CAFs, ECM and vascular remodeling, inducing EMT, and mediating immune evasion	[208, 209]
Ovarian Cancer	Promoting tumor progression by enhancing cancer cell proliferation and neovascularization	[45]
Prostate Cancer	Drives prostate cancer invasiveness and metastasis by inducing EMT and immune suppression	[210]
B-NHL	Promotes the progression and metastasis of B-NHL by inducing EMT and immune escape	[211]
B-cell acute lymphoblastic leukemia (B-ALL)	Promotes the migration and invasion of B-ALL cells by driving the formation of CAFs	[212]
Melanoma	Promotes tumor cell migration and metastatic dissemination by driving tumor-associated angiogenesis and blunting the anti-tumor immunity	[213–215]

#### Small-molecule drugs

TGF-β-targeting small molecules typically function by inhibiting the kinase activity of TGF-β receptors, thereby disrupting the downstream complex signaling network for therapeutic benefits (Fig. 5).

Galunisertib (LY2157299**)** is an oral small molecule inhibitor that specifically inhibits the kinase activity of TβRI to block the phosphorylation of SMAD2, thereby suppressing the activation of the canonical TGF-β signaling pathway. Since TβRI is the common receptor for all three TGF-β ligands (TGF-β1/2/3), Galunisertib can effectively block the signal transduction initiated by these three ligands, and has shown anti-tumor activity in various tumor-bearing animal models, including aggressive B-cell non-Hodgkin lymphoma (B-NHL), breast, colon, and lung cancers as well as HCC [184, 185]. Moreover, Galunisertib synergistically promoted the anti-tumor effect of Doxorubicin by upregulating p-P38 MAPK and inhibiting the TGF-β/SMAD2/3 and PI3K/AKT signaling pathways in B-NHL [185]. Galunisertib greatly enhanced the efficacy of radiotherapy in HNSCC patients by reducing the radio resistance of tumor cells [186]. Likewise, adding Galunisertib to neoadjuvant chemoradiotherapy significantly improved the complete response rates in locally advanced rectal cancer with acceptable safety [187].

Apart from the direct effect on tumor cells, Galunisertib promotes the anti-tumor immunity by mitigating the inhibitory effects of TGF-β inside and outside the TME, reducing disease-aggravating Tregs in TDLNs, and enhancing the immunotherapeutic effect of IL-15-activated DCs [188]. Indeed, when combined with oral probiotic microgel, Galunisertib significantly promoted the anti-tumor immune response in CRC patients [189]. In addition, Nadal E, et al. explored the efficacy and safety of Galunisertib combined with Nivolumab (a PD-1 inhibitor) in the treatment of patients with advanced solid tumors and NSCLC. The results were quite inspiring as the combined treatment was well tolerated, no dose-limiting toxicity occurred, and 24% and 16% of the patients achieved partial remission and stable conditions, respectively [190].

Together, these results imply that Galunisertib has the potential to serve as an adjunctive treatment to enhance the efficacy of chemotherapy, radiotherapy, and/or immunotherapy and to reduce the reliance on radical surgery in tumor patients, albeit more clinical trials are needed to further validate the results and guide its clinical implementation in tumor therapy.

Vactosertib (EW-7197), a second-generation TGF-β inhibitor, exerts its therapeutic effects through dual inhibition of both TβRI (ALK5) and ACVR1B (ALK4), blocking SMAD2/3 phosphorylation and downstream pro-cancer signaling while remodeling the TME [216]. Vactosertib has shown promising effects either as a monotherapy or in combination with chemotherapy/immunotherapy in multiple preclinical and clinical studies. In a CRC model, Vactosertib effectively inhibited the proliferation of CT-26 cells, retarded tumor growth, and promoted tumor necrosis while simultaneously decreasing tumor fibrosis by downregulating the expression of α-SMA and COL1A1 [217]. Vactosertib also inhibited the activity of MMP-9 and increased the expression of CDH1 (E-cadherin), thereby suppressing the invasion of colon cancer cells [217]. In the PDAC model, Vactosertib greatly sensitized chemo-resistant pancreatic tumors to Gemcitabine treatment by decreasing the formation of tumor ECM, resulting in augmentation of Gemcitabine-induced tumor cell apoptosis and inhibition of tumor growth and metastasis in pancreatic cancer patients [218]. In a Phase Ib study, Vactosertib in combination with Oxaliplatin and 5FU/LV (FOLFOX) showed promising efficacy in patients with metastatic pancreatic cancer who had failed first-line Gemcitabine/Nab-paclitaxel treatment [219]. Likewise, Vactosertib exhibited a favorable safety profile, and synergistically promoted the efficacy of Pomalidomide in patients with relapsed/refractory multiple myeloma (RR-MM) in a phase Ib trial (NCT03143985) [220]. Moreover, a recent open-label, multicenter Phase Ib/II study found that the combination of Vactosertib and Imatinib was well tolerated, and exhibited promising clinical activity in treating progressive and advanced desmoid tumors [221].

Notably, although targeting TGF-β signaling may increase the sensitivity of tumor cells to chemotherapy, a recent study revealed that pretreatment of multiple acute myeloid leukemia (AML) cell lines with clinically significant kinase inhibitors, including Galunisertib and Vactosertib, unintentionally target the nucleoside transporter SLC29A1 (ENT1), thereby inducing the complete resistance of AML cells to cytarabine by decreasing its intracellular levels [222].

GFH018 specifically inhibits the kinase activity of TβRI by competitively binding to its ATP (adenosine triphosphate) binding site, thereby inhibiting the proliferation, migration, and invasion of tumor cells [223]. The first-in-human phase I, open-label, multicenter study (NCT05051241) showed a favorable safety profile without cardiac toxicity or bleeding of GFH018 in patients with advanced solid tumors [224]. In patients receiving GFH018 monotherapy, nine (18%) displayed stable disease and one with thymic carcinoma experienced tumor shrinkage [224]. Moreover, in combination with Toripalimab (a humanized PD-1 mAb), GFH018 achieved an objective response rate (ORR) of 26.1%, a disease control rate (DCR) of 43.5%, a median progression-free survival (PFS) of 2.0 months, and a median response duration of 7.6 months in patients with recurrent/metastatic nasopharyngeal carcinoma [225]. As such, GFH018 holds potential in tumor therapy either as a monotherapy or as a combination strategy.

LY3200882 is a novel ATP-competitive inhibitor that selectively targets the serine-threonine kinase domain of TβRI. Preclinical studies have shown that LY3200882 outperforms Galunisertib in terms of pharmacokinetics, pharmacodynamics, and toxicity [226]. LY3200882 upregulated the expression of IFN-β in TAMs within irradiated tumors, which in turn promoted the infiltration of T cells into both the peripheral and core regions of the tumor, thereby enhancing the tumor-killing effects of radiotherapy [227]. LY3200882, when co-delivered with PD-L1 siRNA via a programmed site-specific delivery nanosystem, not only induced the immunogenic cell death of tumor cells and the maturation of DCs, but also synergistically worked with PD-L1 siRNA to enhance the anti-tumor immunity by downregulating the expression of ECM in CAFs, resulting in enhanced influx of effector T cells and nanomedicines in TNBC of mice [228]. Moreover, LY3200882 exhibited a synergistic effect with Gemcitabine in promoting apoptosis while inhibiting the invasion and metastasis of bladder cancer cells both in vitro and in allogeneic tumor models of TNBC [229]. In the first-in-human phase I multicenter study with 139 patients enrolled (NCT02937272), LY3200882 was safe as monotherapy and well tolerated in combination with other anti-cancer reagents, as evidenced by mainly mild, manageable, and reversible treatment-related adverse events (TRAEs) [226]. Notably, the combination therapy comprising LY3200882, Gemcitabine, and Nab-paclitaxel, showed an overall 75% (9/12) DCR in patients with treatment-naive advanced pancreatic cancer. Moreover, LY3200882 as monotherapy induced 18% DCR (7/40) in patients with relapsed grade 4 glioma [226].

YL-13027 can specifically inhibit the activity of TβRI/ALK5 without affecting the functions of other related kinases by competitively binding to TβRI’s ATP-binding site, and thereby suppresses the proliferation, migration, and invasion of tumor cells. In addition, it inhibits the pEMT of tumor cells and regulates the TME to enhance anti-tumor immune responses. A phase I trial on advanced solid tumors (NCT03869632) showed that YL-13027 was well-tolerated and most of the common TRAEs were grade 1–2. Moreover, in patients receiving YL-13027, some TNBC patients achieved partial remission, and some patients had stable disease [230]. In the trial for skull base chordoma, the tumor growth was significantly slowed down in patients receiving YL-13027, with PFS exceeding 6 months, improved symptoms, and good safety [231].

SB-431542 is an inhibitor of the type I receptors of the TGF-β superfamily, including ALK4, ALK5, and ALK7 [232], by competing for the ATP-binding sites, thereby preventing the activation of downstream SMAD proteins [6]. It can induce the phenotypic maturation of both mouse bone marrow-derived DCs (BMDCs) and human DCs, and promotes their ability to activate naive T cells and NK cells, leading to augmented anti-tumor immunity [233]. Moreover, SB-431542 reverses TGF-β-mediated downregulation of MHC-I and restores the antigen presentation by inhibiting the miR-23a-3p/IRF1 axis, thereby enhancing the cytotoxicity of CD8^+^ T cells against HCCA cells [234].

LY2109761 is an orally bioavailable small-molecule pyridine derivative that dually inhibits both TβRI and TβRII. This dual-receptor blockade abrogates SMAD2/3 phosphorylation, and suppresses canonical TGF-β/SMAD signaling transduction. In preclinical studies, LY2109761 induced the apoptosis of multiple human OC cells and osteosarcoma cells, and significantly augmented the cytotoxicity of Cisplatin on these cells in vitro and/or in xenograft mouse models [235, 236]. Recently, Davide Melisi et al. found that LY2109761 effectively inhibited both basal and TGF-β-induced invasiveness and metastatic capacity of pancreatic cancer cells, and induced their anoikis in vitro [237]. In vivo, LY2109761, either alone or together with Gemcitabine, significantly retarded the tumor growth, prolonged the survival, and reduced spontaneous abdominal metastases in athymic mice with orthotopically implanted human pancreatic cancer cells. Mechanistically, the anti-metastatic effect of LY2109761 was attributed to its suppression of TβRI/II kinase activity in tumor cells or host tissue microenvironment [237].

In addition, many other small molecule TGF-β receptor inhibitors, such as SD-208 and SB-505124, have been used in elucidating TGF-β pathway biology and shown significant anti-tumor potential in preclinical studies [238–241]. Nonetheless, these inhibitors, including SB-431542 and LY2109761 as well, have not advanced into late-stage clinical development due to safety considerations and multiple developability challenges like poor metabolic stability, low oral bioavailability, and insufficient aqueous solubility.

#### Monoclonal antibodies (mAbs)

These mAbs directly bind to TGF-β, TβR or other molecules, such as GARP and integrins, to antagonize their function in the activation or transduction of TGF-β signaling (Fig. 5).

SRK-181 is a fully human mAb that selectively binds to latent TGF-β1 with high affinity to inhibit its activation. SRK-181 neither affects the function of human platelets nor induces cytokine release in human peripheral blood [242]. Moreover, comprehensive preclinical studies revealed that SRK-181 was well-tolerated in multiple species, with no observed TRAEs after four-week administration of 200 mg/kg in rats and 300 mg/kg in monkeys [242]. Together, these results indicate that selective blockade of TGF-β1 may avoid the dose-limiting toxicities previously seen with pan-TGF-β inhibitors and thereby may represent a potential target to overcome the primary/acquired resistance in cancer patients receiving checkpoint blocking therapy (CBT). Indeed, coadministration of SRK-181 and an anti-PD-1 antibody in mice harboring syngeneic anti-PD-1-resistant tumors induced profound anti-tumor responses and survival benefit, accompanied with increased intratumoral CD8^+^ T cells and decreased immunosuppressive myeloid cells [243].

TβM1 (LY2382770) is a humanized TGF-β1-specific mAb capable of preventing its binding to cognate receptors. In a Phase I clinical trial involving 18 patients with advanced metastatic cancer, TβM1 showed good tolerance and a long half-life, but its pharmacodynamic effect and anti-tumor activity were insignificant, leading to the discontinuation of treatment after 2 to 4 cycles [244]. The study's limitations include a small sample size, short treatment duration, and high tumor heterogeneity, and thus more trials are needed to assess its potential efficacy in tumor immunotherapy.

NIS793 is a human IgG2 mAb with high affinity for TGF-β1/2 and a slightly lower affinity for TGF-β3. In mouse models of breast cancer and CRC, NIS793 repolarized fibroblasts to an interferon-licensed CAF population and facilitated the infiltration of CD8^+^ T cells into the tumor interior [245]. In the first-in-human study investigating the clinical safety and efficacy of NIS793 plus Spartalizumab (PDR001, a human anti-PD-1 IgG4 mAb) in the treatment of patients with advanced solid tumors (NCT02947165), no dose-limiting toxicities were observed, indicating that NIS793 was well tolerated in combination with Spartalizumab [246]. NIS793 effectively depleted active TGF-β in peripheral blood, and decreased TGF-β target genes while increasing immune-related signatures in paired (before *vs.* after treatment) tumor biopsy samples. Notably, within this heavily anti-PD-1 pretreated patient population (n = 120), partial responses were achieved in one RCC patient and two patients with microsatellite stable CRC (MSS-CRC), and stable disease was observed in 29 patients [246]. Despite the relatively small numbers of patients and samples, this preliminary proof-of-mechanism study supports further explorations of combinational usage of NIS793 and reagents with different action mechanisms in tumor immunotherapy.

Fresolimumab (GC1008), a pioneering humanized pan-TGF-β-neutralizing mAb (IgG4), binds all isoforms of mammalian active TGF-β (TGF-β1/2/3) with high affinity to block ligand-receptor engagement, and thus suppresses the pro-fibrotic, pro-metastatic, and immunosuppressive effects of TGF-β signaling. In a multicenter phase I trial (NCT00356460), Fresolimumab demonstrated acceptable safety at the maximum dose of 15 mg/kg, albeit that 4 out of 29 patients developed benign and reversible keratoacanthomas [247, 248]. Moreover, in 28 patients with advanced malignant melanoma treated with Fresolimumab, one achieved a partial response and six exhibited stable disease with a median PFS of 24 weeks [247]. In addition, patients with metastatic breast cancer receiving the high-dose Fresolimumab during radiotherapy experienced longer median overall survival than the lower dose group, which was associated with increased cell counts and a strikingly boosted CD8^+^ central memory pool in the peripheral blood (NCT01401062) [249, 250].

SAR439459 is a variant of Fresolimumab with a single difference in the Fc region that enhances manufacturability and other properties [251]. In syngeneic tumor models, SAR439459 alone retarded tumor growth, and its combination with anti-PD-1 led to complete tumor regression and augmented anti-tumor immune responses that were evidenced by enhanced proliferation/effector function while reduced exhaustion of intratumoral CD8^+^ T cells [251]. In the phase I/Ib first-in-human study (NCT03192345) evaluating the safety, pharmacokinetics, pharmacodynamics, and anti-tumor activity of SAR439459 plus/minus the PD-1 inhibitor Cemiplimab, SAR439459 monotherapy or combination with Cemiplimab appeared to be relatively safe and tolerable in some patients, but failed to induce sufficient anti-tumor responses in patients with advanced solid tumors [252, 253]. Notably, treatment with SAR439459 induced fatal bleeding in 21.4% of patients with HCC, resulting in the premature termination of this clinical study [252].

LY3022859 (IMC-TR1) is an anti-TβRII human IgG1 mAb. In a phase I clinical trial aiming to determine the dose in patients with advanced tumors (NCT01646203), doses beyond 25 mg/kg were considered to be unsafe as worsening symptoms, mainly uncontrolled cytokine release, appeared despite prophylaxis [254]. In animal models, the murine equivalent of the human LY3022859 mAb (MT1) unleashed IFN-β expression in TAMs within irradiated tumors, and thereby promoted the infiltration of T lymphocytes, leading to enhanced radiotherapy efficacy [227]. The safety and efficacy of combined therapy comprising LY3022859 and radiation in human tumor patients warrant further investigation.

mAbs targeting GARP and/or the GARP-latent TGF-β complex. Given the role of GARP in the activation of latent TGF-β (Fig. 1), it is conceivable that antibodies blocking the release of active TGF-β from the GARP-latent TGF-β complex will repress the initiation of TGF-β signaling and augment the anti-tumor immunity. Indeed, the murine anti-human GARP (PIIO-1) that specifically binds to the ligand-free GARP at the latent TGF-β1 interacting domain and thus blocks the complex formation, disrupts the canonical TGF-β signaling, enhances the infiltration and effector function of CD8^+^ T cells in the TME, and overcomes the primary resistance to anti-PD-1 therapy in mice with MB49 bladder cancer, Lewis Lung Carcinoma (LLC), and CMT-167 lung cancer [255]. Notably, PIIO-1 does not cause thrombocytopenia as it shows no binding to the GARP-latent TGF-β complex on platelets, thus minimizing bleeding risks often associated with pan-TGF-β blocking. DS-1055a, a novel afucosylated anti-human GARP mAb (human IgG1), was capable of directly depleting adult T-cell leukemia/lymphoma (ATL) in vitro and retarding tumor growth indirectly by eliminating suppressive GARP^+^ Tregs in the TME of humanized mice bearing HT-29 cells [256, 257]. The first-in-human phase I clinical trial (NCT03821935) revealed that Livmoniplimab (ABBV-151), a first-in-class human IgG4/ҡ mAb binding to the human GARP-TGF-β1 complex, was well-tolerated in patients with advanced solid tumors in the dose-escalation phase. Despite that no objective responses were observed in patients receiving Livmoniplimab alone, Livmoniplimab and Budigalimab (an anti-PD-1 mAb) combination therapy led to a 15% ORR with median response duration of 8.4 months in these heavily pretreated patients [258]. BPB-101 is a novel bispecific, trifunctional antibody that binds to the GARP-TGF-β complex and/or small latent complex (SLC), active TGF-β, and PD-L1, and is capable of effectively blocking both the TGF-β and PD-1/PD-L1 signaling pathways [259]. BPB-101 exhibited a favorable safety profile in non-human primate toxicity studies, and achieved a 50% complete tumor elimination rate at the dose of 5 mg/kg in C57BL/6-hGARP mouse bearing MC38-hPD-L1 tumors [259]. Thus, the efficacy of mAbs targeting GARP and/or GARP-TGF-β complex as a monotherapy or in combination with different ICB agents needs to be further developed. Currently, a phase I/II clinical study evaluating the safety, pharmacokinetics and efficacy of BPB-101 as monotherapy in patients with advanced malignant solid tumors is ongoing and expected to be completed soon (NCT05869240).

#### Antisense oligonucleotides (AS-ODNs) and AS-ODNs-modified vaccines

Oligonucleotide therapeutics represent an emerging class of pharmacological agents composed of chemically modified or unmodified short nucleic acid molecules. This category encompasses antisense oligonucleotides (AS-ODNs), small interfering RNAs (siRNAs), microRNAs (miRNAs), aptamers, and DNAzymes. These therapeutic modalities exert their effects through Watson–Crick base pairing with target mRNAs, enabling mechanisms such as gene silencing, steric blockade of translation, and splicing modulation [260]. To block/repress the activation of TGF-β signaling pathway, AS-ODNs directly bind to the mRNAs of TGF-β ligands (such as TGF-β1/2), receptors (such as TβRII) or key downstream molecules (like SMAD7) through base complementary pairing, thereby promoting their degradation or inhibiting their translation (Fig. 5). Compared to traditional small-molecule drugs or mAbs, AS-ODNs offer advantages in terms of target specificity, long-lasting effect, and multifunctionality [261].

Trabedersen (AP12009 or OT-101) is an 18-mer thiophosphate oligodeoxynucleotide that inhibits the expression of human TGF-β2 by complementing a specific region of its mRNA. By using primary tumor cell cultures from patients with high-grade glioma, pancreatic cancer, malignant melanoma and CRC or relevant cell lines, studies have shown that Trabedersen significantly reduced the secretion of TGF-β2, inhibited the proliferation and migration of tumor cells, and reversed TGF-β2-mediated immunosuppression [262]. In a randomized, open-label phase IIb study, convection-enhanced intratumoral delivery of Trabedersen increased the long-term survival rate, but not the short-term tumor control, in high-grade glioma patients compared with conventional chemotherapy, accompanied with fewer side effects [263]. Moreover, Trabedersen treatment upregulated IL-8 and IL-15 production, and prolonged the overall survival in young PDAC patients [264, 265].

ISTH1047 and ISTH0047 are two novel 14-mer and 17-mer phosphorothioate-locked nucleic acid (LNA)-modified AS-ODN gapmers, targeting TGF-β1 and TGF-β2, respectively. Systemic administration of ISTH1047 or ISTH0047 significantly inhibited tumor growth, metastasis and prolonged the survival in xenogeneic and/or syngeneic models of glioma, RCC, breast and lung cancers, which was associated with reduced TGF-β1/2 expression and SMAD2 phosphorylation while increased anti-tumor immunity [266, 267]. Given the minimal off-target effects conferred by the highly specific LNA-modified design, favorable safety profile and profound anti-tumor potential in preclinical studies, these two TGF-β targeting AS-ODNs represent a novel strategy to treat tumor patients.

TGF-β antisense gene-modified vaccines. Besides the TGF-β targeting AS-ODNs, researchers have developed a few TGF-β antisense gene-modified tumor cell vaccines to increase the immunogenicity and enhance the anti-tumor immune response by repressing TGF-β expression (Fig. 5). Belagenpumatucel-L (Lucanix) is an allogeneic tumor vaccine consisting of four irradiated NSCLC cell lines harboring TGF-β2 antisense gene plasmids [268]. In a Phase II clinical trial comprising 75 patients with stage II-IV NSCLC (NCT01058785), Belagenpumatucel-L was well tolerated and its high dose achieved a 15% partial response rate and significantly increased the 1–2 year survival probabilities in 61 late-stage (IIIB and IV) patients with accessible data, likely owing to the increased humoral and cellular immune responses [268]. However, no differences in the overall or PFS rate were observed in patients treated with Belagenpumatucel-L *vs.* placebo as a maintenance therapy in a later phase III study with larger numbers of stage III/IV NSCLC patients who did not progress after platinum-based chemotherapy (NCT00676507) [269]. Nonetheless, further analysis suggested that Belagenpumatucel-L may benefit patients who recently received chemotherapy or radiotherapy [269]. Notably, vaccinations with autologous tumor cells expressing TGF-β2 AS-ODNs significantly prolonged the survival in patients with glioma and other solid tumors [270, 271].

FURIN antisense gene-modified vaccines. Vigil (Gemogenovatucel-T or FANG) is an autologous tumor cell vaccine that is genetically engineered to express GM-CSF (granulocyte–macrophage colony-stimulating factor) and a bifunctional short-hairpin RNA (shRNA) targeting FURIN convertase (Fig. 5), a key protease involved in TGF-β1/2 activation [272]. In a Phase I trial with advanced solid cancers, intradermal vaccinations with ≥ 4 Vigil significantly prolonged the survival of patients, albeit that a trend was noted in patients who received < 4 vaccinations as well [273]. Likewise, in a following randomized, double-blind, and placebo-controlled phase IIb trial in stage III/IV OC (NCT02346747), Vigil as a maintenance treatment significantly increased the overall and relapse-free survival of homologous recombination proficient (HRP) OC patients [274, 275]. No serious TRAEs were observed in these two studies and the common grade 1–2 adverse events were local rejections at the injection site, and Vigil-responsive patients displayed enhanced anti-tumor immune responses [273]. Moreover, in a recent pilot study (NCT02725489), combination therapy of Vigil plus Durvalumab showed promising clinical efficacy in women with relapsed BRCA-wt TNBC or OC [276].

In addition, other AS-ODNs targeting key molecules, such as Mongersen (GED-0301, an oral SMAD7 AS-ODN) showed promising results in phase I and phase II trials in patients with active Crohn's disease, but failed in a further phase III study (NCT02596893) due to a lack of clinical benefit [277]. Hence, further studies are warranted to evaluate the clinical efficacy of these AS-ODNs in patients with various disorders.

#### Dual-functional fusion proteins or bispecific antibodies

The dual-functional fusion proteins normally comprise an antibody targeting the immune checkpoint molecules (like CTLA-4, PD-1 or PD-L1) fused to the ectodomain of TβRII, and thus function as both an ICB and a TGF-β trap to block these two signaling pathways simultaneously (Fig. 5). In preclinical studies, the anti-CTLA-4-TβRII or anti-PD-L1-TβRII fusion proteins exhibited better anti-tumor efficacy as compared to their parent ICBs [278].

Bintrafusp alfa (M7824), a first-in-class bifunctional fusion protein composed of the extracellular domain of human TβRII fused to a human IgG1 anti-PD-L1 mAb, demonstrated differential clinical efficacy across various tumor types. In a Phase I clinical trial (NCT02517398), Bintrafusp alfa showed some clinical activity and manageable tolerability in Sorafenib failed/intolerant advanced HCC patients [279], heavily pretreated patients with CRC [280], esophageal adenocarcinoma [281] or advanced squamous cell carcinoma of the head and neck (SCCHN) [282], and NSCLC patients pretreated with platinum or resistant/refractory to ICB immunotherapy [279, 283–285]. Moreover, in the phase I (NCT02517398, NCT04432597 and NCT04287868) and phase II (NCT03427411) clinical studies, Bintrafusp alfa either as a monotherapy or as a combination therapy with HPV-targeted vaccines (such as PRGN-2009 and PDS0101) showed a manageable safety profile, induced a promising anti-tumor activity and increased the overall survival rate in both ICB-naive and -resistant patients with human papillomavirus (HPV)-associated cancers [286–288]. In newly diagnosed HPV-negative HNSCC patients (NCT04247282), pretreatment with Bintrafusp alfa as a neoadjuvant immunotherapy before surgery enhanced neoepitope-specific tumor T-cell immune responses and reduced myeloid cell tumor infiltration [289]. In line, the combination therapy of Tri-Ad5 vaccine and Bintrafusp alfa achieved clinical-pathological remission in 2 out of 6 patients (33.3%) with a 2-year recurrence-free survival (RFS) rate of 83.3% [290]. The combination therapy of Bintrafusp alfa with a TIGIT inhibitor (M6223) in solid tumors showed a manageable safety profile as well (NCT04457778) [291], but its clinical efficacy in tumor immunotherapy warrants further investigation. The most common TRAEs associated with Bintrafusp alfa were anemia, bleeding, fatigue, eczema, pruritus, and increased amylase.

It was reported that Bintrafusp alfa induced a response rate as high as 85.7%, significantly higher than Pembrolizumab (an anti-PD-1 mAb), in heavily pretreated PD-L1^high^ NSCLC patients [283]. However, Bintrafusp alfa monotherapy or Bintrafusp alfa combined with concurrent chemoradiotherapy (cCRT) followed by Bintrafusp alfa failed to exhibit superior efficacy to Pembrolizumab alone or placebo plus cCRT followed by Durvalumab (an anti-PD-L1 mAb) in unresectable stage III or advanced PD-L1^high^ NSCLC patients in two recent phase II (NCT03840902) and phase III (NCT03631706) studies [292, 293]. Moreover, although Bintrafusp alfa as a second-line therapy appeared to have clinical activity in a phase I trial (NCT02699515) in pre-treated Asian patients with biliary tract cancer [294, 295] or recurrent/refractory gastric cancer [296], and in a phase II study with locally advanced/metastatic biliary tract cancer patients (NCT03833661) [297], its combination with gemcitabine plus cisplatin (GemCis) did not exhibit a clinically meaningful benefit compared with GemCis alone as first-line treatment for biliary tract cancer patients in the phase II/III trial (NCT04066491) [298]. In a recent phase II single-arm clinical trial (NCT05005429), limited clinical efficacy of Bintrafusp alfa was observed in patients with advanced pleural mesothelioma [299]. Thus, it appears that Bintrafusp alfa might be more effective than PD-1/PD-L1 single blockers in heavily pre-treated patients, but no superior efficacy was achieved when used as first-line therapy.

Likewise, SHR-1701 is a bifunctional protein containing a mAb against PD-L1 fused with the extracellular domain of TβRII [300]. In preclinical studies, SHR-1701, but not anti-PD-1 antibodies, enhanced the proliferation and function of CD8^+^ T cells in mice bearing CMT167 lung cancer pretreated with chemotherapy, suggesting that SHR-1701 might be able to reinvigorate the anti-tumor immune response in lymphopenic cancer patients after chemotherapy [300]. In two phase I studies, SHR-1701 showed an encouraging anti-tumor activity and acceptable safety profile in pretreated patients with gastric cancer (NCT03710265) or metastatic cervical cancer (NCT03774979), particularly those with increased TGF-β signaling and/or PD-L1 expression [301, 302]. In a phase II trial (NCT04580498), neoadjuvant SHR-1701 plus chemotherapy, followed by surgery or radiotherapy, exhibited a promising efficacy and controllable side effects in unresectable late-stage NSCLC patients [303]. Moreover, clinical data showed that a regimen combining SHR-1701 and Famitinib (a multi-targeted tyrosine kinase inhibitor) may represent an effective and safe subsequent-line therapy for refractory advanced PDAC, biliary tract cancer, and gallbladder cancer patients [304, 305]. Likewise, SHR-1701 in combination with Bevacizumab (an anti-VEGFA mAb) and XELOX (Capecitabine plus Oxaliplatin) induced potent anti-tumor immunity in a phase II/III trial with unresectable metastatic CRC patients (NCT04856787) [306]. The main TRAEs related to SHR-1701 include increased gamma-glutamyltransferase or aspartate aminotransferase, anemia, hyponatremia and rash.

In addition, BiTP is an IgG1/IgG2 hybrid bispecific antibody targeting pan-TGF-β and human PD-L1 [307]. In preclinical studies, BiTP not only showed superior anti-tumor activity as compared to anti-PD-L1 or anti-TGF-β monotherapy in a murine TNBC model [307], but also synergized with chemotherapy in neoadjuvant settings to retard tumor growth while promoting anti-tumor immunity in a murine PDAC model [307, 308]. Nonetheless, the clinical safety and efficacy of BiTP in various solid tumors requires further investigation. 

4 T-Trap is an innovative protein comprising the ectodomain of TβRII fused to Ibalizumab (a non-immunosuppressive human IgG4 anti-CD4 mAb), thereby conferring the ability to specifically block TGF-β signaling in CD4^**+**^ T cells. In preclinical mouse tumor models, 4 T-Trap reorganized tumor vasculature, induced tumor tissue hypoxia, and promoted VEGF expression and cancer cell death in an IL-4-dependent manner. Moreover, combined VEGF inhibition augmented the anti-tumor effect of 4 T-Trap [309]. Therefore, targeted repression of TGF-β signaling in CD4^+^ T cells may elicit an effective anti-tumor immune response by reshaping the TME while minimizing the potential adverse effects associated with systemic TGF-β blockade [309].

In summary, despite that many preclinical and clinical studies have shown the therapeutic potential of TGF-β inhibitors in the treatment of cancer patients, numerous clinical trials, as summarized in Table 3, are still ongoing at present to further confirm the safety and efficacy of different TGF-β-targeting agents.

**Table 3:** Ongoing clinical trials of TGF-β targeted agents in tumor patients

Agents	Strategies	Targets	Tumor types	Clinical trials
Galunisertib (LY2157299)	Small-molecule kinase inhibitor	TβRI	Rectal Cancer Advanced CRC Prostate Cancer	NCT02688712 NCT05700656 NCT02452008
Vactosertib (EW-7197)	Small-molecule kinase inhibitor	TβRI	Esophageal Adenocarcinoma Progressive Osteosarcoma	NCT06044311 NCT05588648 NCT05400122
YL-13027	Small-molecule kinase inhibitor	TβRI	Refractory Metastatic Pancreatic Cancer	NCT06199466
SAR439459	mAb	Pan-TGF-β	Plasma Cell Myeloma	NCT04643002
DS-1055a	mAb	TGF-β1	Advanced Cancer, Metastatic Solid Tumor	NCT04419532
Livmoniplimab (ABBV-151)	mAb	GARP-TGF-β1 complex	Urothelial Carcinoma	NCT06632951
Trabedersen (OT-101)	AS-ODNs	TGF-β2 mRNA	NSCLC	NCT06579196
Bintrafusp alfa (M7824)	Bifunctional fusion protein	TGF-β and PD-L1	Thymoma and Thymic Carcinoma Advanced Sarcoma Advanced Intrahepatic Cholangiocarcinoma Metastatic Non-Prostate Genitourinary Malignancies HPV-Associated Malignancies Small Bowel and Colon Cancers	NCT04417660 NCT04874311 NCT04708067 NCT04235777 NCT04708470
SHR-1701	Bifunctional fusion protein	TGF-β and PD-L1	Advanced Rectal Cancer Lung Squamous Cell Carcinoma Gastric or Gastroesophageal Cancer Advanced Solid Tumors and B-cell Lymphomas Classical Hodgkin Lymphoma	NCT05300269 NCT04937972 NCT04950322 NCT04407741 NCT05896046
Vigil	AS-ODNs-modified vaccine	FURIN	Ovarian Cancer	NCT02346747

### Challenges of TGF-β-targeting therapy

#### Dual roles of TGF-β in tumor development and progression

As depicted in Fig. 2, TGF-β exhibits complex dual roles in cancer initiation, development and progression. In the early tumorigenic phase, TGF-β primarily exerts tumor-suppressive effects by inhibiting cell proliferation and inducing apoptosis [85]. Conversely, in the advanced tumor progression stage, TGF-β mainly adopts a pro-tumorigenic role by promoting the EMT, metastasis, immune evasion, and remodeling of the TME [310]. The paradoxical roles of TGF-β during distinct stages of tumor development pose formidable challenges for designing targeted therapies. An ideal TGF-β-targeting agent must achieve spatiotemporally confined inhibition of TGF-β expression and signaling in advanced tumors to suppress tumor growth, metastasis, and therapy resistance, and promote anti-tumor immune responses, while concurrently preserving its tumor-suppressive functions in normal tissues and early-stage tumors. Failure to maintain this balance risks abrogating tumor suppression or even accelerating carcinogenesis [311].

#### Systemic toxicity

Given the critical roles of TGF-β in maintaining tissue homeostasis, such as skin proliferation, immune homeostasis, and vascular function, TGF-β-targeted drugs often exhibit multi-organ toxicity that is commonly associated with the specificity and mechanism of drugs as well as the dose and administration route [6].

The TGF-β family consists of three highly homologous isoforms, TGF-β1/2/3, which exhibit distinct expression patterns, biological functions, and disease-specific roles. Early pan-TGF-β inhibitors (e.g., Fresolimumab and Galunisertib), which concomitantly block all TGF-β isoforms, disrupt vascular homeostasis and platelet function, thus leading to severe adverse effects such as hemorrhage and cardiotoxicity [247, 249, 312]. In contrast, selective TGF-β1 inhibitors retain the physiological functions of TGF-β2/3 and exhibit reduced systemic toxicity. For instance, in a 4-week rat toxicology study, the selective inhibitor SRK-181 did not induce cardiac valvulopathy, a dose-limiting toxicity previously reported with pan-TGF-β inhibitors [242]. Thus, further investigations on the expression and function of each TGF-β isoform in various tumor types are needed to guide the clinical application of isoform-specific TGF-β inhibitors, which will enhance the therapeutic efficacy while minimizing adverse effects in patients.

Moreover, the administration route significantly shapes the toxicity profile of TGF-β inhibitors by modulating the systemic exposure and tissue distribution. In general, intravenous delivery facilitates rapid dissemination of drugs to multiple organs and frequently precipitates systemic adverse effects, while subcutaneous administration commonly attenuates acute toxicity by slowing drug absorption and avoiding sharp peaks in plasma concentration. For instance, high-dose intravenously-administered Galunisertib accumulates in cardiac valves, causing hemorrhagic and degenerative valvulopathies [313]. By contrast, subcutaneously-administered AVID200 showed absence of infusion-related reactions (e.g., fever and chills) and a 25% reduction in dose-limiting toxicities such as cutaneous keratopathy compared to the intravenous delivery in a phase I trial [313].

These findings highlight the systemic challenges of TGF-β inhibition and underscore the need for cautious translation from preclinical models to clinical practice. Hence, effective toxicity management remains essential for patients receiving TGF-β-targeted therapies.

## Approaches to improve TGF-β pathway targeted therapy

### Drug combination therapy strategy

As discussed above, TGF-β-targeted agents often enhance the anti-tumor efficacy through multimodal synergism with chemotherapy, radiotherapy, and immunotherapy in cancer patients. TGF-β inhibitors reverse key mechanisms of drug resistance, including EMT, cell cycle arrest, and expression of anti-apoptotic proteins, mediated by TGF-β signaling. For instance, the TβRI inhibitor Galunisertib suppresses SMAD2/3 phosphorylation and downregulates molecules involved in MDR and DNA repair pathways, thereby increasing tumor cell apoptosis by threefold in combination with Paclitaxel in murine TNBC models [184, 314]. Radiotherapy directly damages the DNA and induces the death of tumor cells through high-energy radiation, leading to the release of tumor antigens and chemokines as well as the activation of TGF-β signaling [315]. Moreover, radiotherapy-induced fibrotic stroma forms a physical barrier that impedes drug delivery and immune cell infiltration. Combining TGF-β signaling inhibitors with radiotherapy multidimensionally reverses the immunosuppressive state of the TME by promoting the tumor antigen presentation while reducing collagen deposition and stromal stiffness, thereby improving tumor tissue perfusion and facilitating immune cell infiltration [316, 317]. Accordingly, preclinical studies have demonstrated that the combination of anti-αvβ8 antibody (ADWA-11) with Gemcitabine and radiotherapy significantly reduced tumor invasion and metastasis while enhancing radiosensitivity in pancreatic cancer models [318]. Furthermore, combinatorial immunotherapy approaches have emerged as viable cancer therapeutic strategies for improving patient responses and outcomes [319]. Numerous preclinical and clinical studies have reported augmented anti-tumor activity when TGF-β inhibitors, such as SAR439459, NIS793, Fresolimumab, PIIO-1 and SRK-181, are used in combination with anti-PD-1/PD-L1 agents [243, 246, 247, 249, 252, 255, 320]. Of note, GFH018 in combination with Toripalimab (anti-PD-1) exhibited manageable toxicity and durable anti-tumor activity in patients with recurrent or metastatic nasopharyngeal carcinoma [225].

### Biomarker monitoring

#### Biomarkers for patient stratification

TGF-β signaling components have emerged as promising biomarkers for patient stratification due to their prognostic and predictive values. Elevated TGF-β levels in primary tumors and plasma are associated with poor prognosis in patients with CRC and pancreatic cancer [321–324]. Moreover, mRNA expression levels of SKIL and PMEPA1 are positively correlated with TGF-β1 mRNA expressions in HCC tissues, and can be significantly downregulated by the TβR blocker Galunisertib. As such, expression of SKIL and PMEPA1 can be used as a biomarker to select HCC patients for Galunisertib treatment [325]. Furthermore, mRNA expression levels of TGF-β and SMAD2 in platelets are related to local tumor and pathological features, and thus have been used as potential biomarkers for the diagnosis and grading of colon cancer patients [326].

#### Biomarkers for reaction monitoring

Beyond patient stratification, measurement of TGF-β pathway components in blood, serum, and tissues represents a rapid, accurate, and inexpensive approach to predict therapeutic response in patients treated with TGF-β signaling blockers [319]. For instance, evaluating changes in SMAD2/3 phosphorylation levels in tumor cells serves as a direct indicator of TGF-β pathway activity and a predictive marker for therapeutic efficacy in cancer patients treated with Bintrafusp alfa, Abituzumab, or Galunisertib [313, 320, 327]. Similarly, Fresolimumab therapy response positively correlates with the decrease in blood TGF-β1 levels, and thus the latter represents a measurable parameter for target engagement [328]. Furthermore, Trabedersen exerts its inhibitory effect by downregulating TGF-β2 mRNA. Consequently, changes in TGF-β2 mRNA levels during treatment may serve as a biomarker for monitoring therapeutic response [328].

### Novel strategy targeting TGF-β

#### Matrix-targeted therapeutic strategies

ECM components like integrins play a pivotal role in the activation of TGF-β (Fig. 1). Consequently, targeting integrins holds potential in blocking TGF-β signaling. GLPG-0187 and Cilengitide (EMD121974), which are respectively a pan integrin and a potent αvβ3/αvβ5 integrin small-molecule inhibitor, may block the activation of TGF-β and thus retard tumor growth and/or enhance the tumor-eradicating ability of T cells [6]. However, Cilengitide failed to demonstrate significant therapeutic efficacy in multiple tumor clinical trials [6]. Intriguingly, Abituzumab (DI17E6), a human IgG2/ҡ mAb against pan-αv integrins, appeared to increase the overall survival rate in a phase I/II trial on KRAS wild-type metastatic CRC (NCT01008475), especially in patients with high αvβ6 expression [320]. Moreover, Abituzumab lowered the incidence of bone lesion progression, despite the comparable PFS rate, in a randomized, double-blind phase II study on chemotherapy-naive patients with metastatic castration-resistant prostate cancer (NCT01360840), likely via directly inhibiting the migration and invasion of cancer cells [317, 329]. 264RAD and 130H2 are two other human IgG mAbs binding to αvβ6/αvβ8 or the β8 subunit, respectively [330, 331]. Preclinical studies have shown that 264RAD effectively inhibited the growth/metastasis of Detroit 562 cells, multiple human breast cancer cells, and human PDAC in xenograft models by targeting αvβ6/αvβ8 integrin, thereby disrupting TGF-β activation and other integrin-mediated signaling pathways [330, 332–334]. Likewise, 130H2, either alone or in combination with PD-1 suppression, significantly retarded the growth of mammary carcinoma EMT6 cells and colon carcinoma CT26 cells in syngeneic mouse models by reversing TGF-β-mediated immunosuppression, thereby modulating macrophage polarization and enhancing immune cell infiltration [331].

In addition, given that TGF-β drives tumor progression, fibrosis, and therapy resistance partially by activating CAFs and promoting aberrant ECM production/deposition in tumor tissues, it is conceivable that hampered TGF-β signaling would lead to reduced ECM production and decreased stiffness of tumor tissues, and thereby promoting the immune cell infiltration and drug penetration, eventually increasing the efficacy of chemotherapy or immunotherapy in clinic practice [6, 318, 335]. Hence, agents targeting the ECM-TGF-β axis represent a novel targeting strategy for tumor therapy.

#### Nanodelivery systems targeting TGF-β

Nanoparticles utilize the enhanced permeability and retention (EPR) effect for passive targeting and can be functionalized with targeting ligands (e.g., peptides and antibodies) to specifically target tumor cells or stromal components [336]. Various TGF-β-targeted nanodelivery systems have demonstrated significant therapeutic potential. For example, disulfide-crosslinked PEG-PDMAEMA nanoparticles (122 nm) co-delivering TGF-β1 siRNA and FOXM1 siRNA resulted in approximately 67%/83% reduction in tumor volume/lung metastases, respectively, in murine TNBC models without hepatotoxicity [337]. The amphipathic hydroxyethyl starch-polycaprolactone (HES-PCL) nanoparticles-based targeted delivery of TGF-β inhibitor LY2157299 to the tumor site enhanced CAR-T cell therapy efficacy in lymphoma models [338]. A nanoemulsion system (SSB NMs) co-delivering selenocystine and LY2157299 into tumor cells enhanced NK cell-mediated tumor killing via concurrent NKG2DL upregulation and TGF-β/SMAD inhibition in tumor cells [339]. The HLP@SiTGF-β1 hybrid nanoparticle, containing TGF-β1-targeting siRNA and hybrid membrane fusion liposomes (loaded with the phototherapeutic agent IR-780) with Lewis lung carcinoma cell membranes, effectively penetrated the blood–brain barrier and suppressed the intracranial tumor growth in lung metastasis models partially by inhibiting TGF-β and promoting anti-tumor immunity [340]. Additionally, gelatinase-responsive nanoparticles (GPNPs) co-loaded with anti-PD-1 and Galunisertib significantly inhibited tumor progression and prolonged the survival of mice in multiple lung cancer models [341]. Collectively, these nanoplatforms overcome the limitations of TGF-β therapies through improved targeting, controlled release, reduced adverse effects and enhanced synergistic effects in remodeling the immunosuppressive TME and inhibiting tumor growth in preclinical studies. Nonetheless, the safety and efficacy of TGF-β-targeting nanodelivery systems in patients warrant further investigation.

## Concluding remarks

In this review, we systematically summarized and discussed the multifaceted roles of TGF-β in tumorigenesis and cancer progression, along with the advances and challenges in TGF-β-targeted therapeutics in cancer treatments. As a pleiotropic cytokine with diverse functions within and beyond the immune system, TGF-β exhibits both pro- and anti-tumor activities depending on the dose, context, as well as the type and stage of tumors. This functional switch is orchestrated by multidimensional mechanisms, including the genetic mutations and abnormal regulation of signaling pathways in tumor cells as well as the complex interactions among stromal cells, cancer cells, immune components, and ECM remodeling within the TME. In general, TGF-β appears to generate a microenvironment fostering the tumor growth, invasion and dissemination while repressing the anti-tumor responses in most cases during tumor progression, although an anti-tumor effect has been observed in advanced cancers as well [342]. Thus, blockade of the hyperactivated TGF-β signaling pathway holds promise in reversing the immunosuppressive TME to reinvigorate endogenous immune cells and potentiate the anti-tumor immunity.

Currently, the field of TGF-β-targeted therapy is rapidly advancing, with a diverse array of therapeutic modalities, including small molecule inhibitors, mAbs, AS-ODNs, and innovative bifunctional fusion proteins or bispecific mAbs, which have demonstrated considerable potential in cancer therapy. Moreover, both preclinical and clinical studies indicate that combining TGF-β-targeted agents with chemotherapy, radiotherapy, or immunotherapy may synergistically enhance anti-tumor efficacy and overcome treatment resistance, thereby substantially improving the overall therapeutic outcomes [4, 5].

Despite considerable progresses, critical barriers impede the clinical translation of TGF-β-targeted therapies. Given the essential roles of TGF-β family in various cellular and physiological processes, how to effectively counteract the pathological functions of TGF-β without affecting its physiological roles represents a primary challenge of TGF-β-targeted therapy in the clinic. For instance, systemic administration of pan-TGF-β inhibitors often results in severe TRAEs like multiorgan toxicity in patients [6]. Moreover, another challenge is the lack of precise, individualized biomarkers to guide the application of TGF-β inhibitors in patients displaying great interpatient heterogeneity in tumor types, mutational spectra, and TGF-βsignaling activities along tumor progression. Thus, it is conceivable that the lack of biomarker-guided stratification of cancer patients mainly accounts for the promising efficacy of anti-TGF-β therapy in a small but unpredictable subset of patients while concurrently preventing its overall clinical beneficial effects in large clinical trials [6]. Finally, the dynamic crosstalk/regulation among TGF-β and other signaling pathways in the TME remains largely unexplored. This knowledge gap poses challenges for clinical trials, particularly in the rational design of combination therapeutic strategies, ultimately compromising the accuracy of treatment response evaluation.

Therefore, future efforts may focus on the following directions to increase the efficacy and reduce the adverse effects of TGF-β-targeting therapy in tumor patients: 1) further elucidating the distinct functions and molecular mechanisms of different TGF-β isoforms in the initiation, development and progression of various tumor types; 2) developing isoform-selective TGF-β inhibitors with increased specificity/affinity and novel programmed site/cell-specific delivery systems, like the nanosystem invented by Huo’s group [228], to enhance the inhibitory effect in desired sites/cells while reducing the potential systemic toxicity; 3) identifying dynamic/precise biomarkers for patient stratification, for instance by monitoring the aberrant TGF-β signaling activity via liquid biopsies or screening for genetic mutations, to guide the tailored implementation of appropriate TGF-β blocking strategies in patients; and 4) systematically deciphering the complex crosstalk between TGF-β signaling and other pathways in the TME, which may provide a theoretical foundation for rational combination therapy design, refine clinical trial strategies, and enable accurate assessment of therapeutic potential.

## Data Availability

Not applicable.
